# Pharmacological or TRIB3-Mediated Suppression of ATF4 Transcriptional Activity Promotes Hepatoma Cell Resistance to Proteasome Inhibitor Bortezomib

**DOI:** 10.3390/cancers13102341

**Published:** 2021-05-12

**Authors:** Tiit Örd, Daima Örd, Minna U. Kaikkonen, Tõnis Örd

**Affiliations:** 1 Institute of Genomics, University of Tartu, Riia 23b, 51010 Tartu, Estonia; tiit.ord@ut.ee (T.Ö.); daima.ord@ut.ee (D.Ö.); 2 A.I. Virtanen Institute for Molecular Sciences, University of Eastern Finland, P.O. Box 1627, 70211 Kuopio, Finland; minna.kaikkonen@uef.fi

**Keywords:** integrated stress response, proteasome inhibitor, chemotherapy resistance, Tribbles, cell death

## Abstract

**Simple Summary:**

Proteasome inhibitors are currently used in the treatment of certain blood cancers, and clinical trials to treat solid tumors, including liver cancer, have also been conducted. However, different malignancies are not equally susceptible to proteasome inhibitors, and resistance to the drug may develop during the therapy. Here, we characterize the molecular mechanisms underlying the resilience of liver cancer cells to the proteasome inhibitor bortezomib. The results demonstrate that the activity of the eIF2α–ATF4 stress response pathway affects the viability of cells treated with bortezomib. We found that the pseudokinase TRIB3, an endogenous regulator of ATF4 and a gene highly expressed in liver cancer, resides predominantly at the same chromatin sites as ATF4 and constrains ATF4 activity. The survival of bortezomib-exposed hepatoma cells proved sensitive to TRIB3 overexpression and inactivation. Thus, TRIB3 is a novel factor contributing to bortezomib resistance of liver cancer cells.

**Abstract:**

The proteasome is an appealing target for anticancer therapy and the proteasome inhibitor bortezomib has been approved for the treatment of several types of malignancies. However, the molecular mechanisms underlying cancer cell resistance to bortezomib remain poorly understood. In the current article, we investigate how modulation of the eIF2α–ATF4 stress pathway affects hepatoma cell response to bortezomib. Transcriptome profiling revealed that many ATF4 transcriptional target genes are among the most upregulated genes in bortezomib-treated HepG2 human hepatoma cells. While pharmacological enhancement of the eIF2α–ATF4 pathway activity results in the elevation of the activities of all branches of the unfolded protein response (UPR) and sensitizes cells to bortezomib toxicity, the suppression of ATF4 induction delays bortezomib-induced cell death. The pseudokinase TRIB3, an inhibitor of ATF4, is expressed at a high basal level in hepatoma cells and is strongly upregulated in response to bortezomib. To map genome-wide chromatin binding loci of TRIB3 protein, we fused a Flag tag to endogenous TRIB3 in HepG2 cells and performed ChIP-Seq. The results demonstrate that TRIB3 predominantly colocalizes with ATF4 on chromatin and binds to genomic regions containing the C/EBP–ATF motif. Bortezomib treatment leads to a robust enrichment of TRIB3 binding near genes induced by bortezomib and involved in the ER stress response and cell death. Disruption of TRIB3 increases C/EBP–ATF-driven transcription, augments ER stress and cell death upon exposure to bortezomib, while TRIB3 overexpression enhances cell survival. Thus, TRIB3, colocalizing with ATF4 and limiting its transcriptional activity, functions as a factor increasing resistance to bortezomib, while pharmacological over-activation of eIF2α–ATF4 can overcome the endogenous restraint mechanisms and sensitize cells to bortezomib.

## 1. Introduction

Liver cancer is the seventh most frequent cancer and the third leading cause of cancer-associated mortality in the world, representing a considerable need for improved treatment [1]. The proteasome, a cytoplasmic and nuclear multi-subunit protein complex that degrades damaged, misfolded or short-lived proteins, is known to play a role in facilitating malignant growth and is considered a target for anti-cancer drugs [2]. The proteasome inhibitor bortezomib has been approved by FDA for use in the treatment of multiple myeloma and mantle cell lymphoma, and a number of phase I or II clinical trials have been carried out to explore the potential of bortezomib in other types of cancer, including solid tumors such as hepatocellular carcinoma (reviewed in [3]). However, as different malignancies have not proven equally sensitive to bortezomib, and resistance to the drug may develop over the course of therapy, solid tumors have not proven particularly amenable to proteasome inhibitor therapy [4]. Therefore, a better understanding of the molecular mechanisms underlying bortezomib resistance is necessary.

Cells exposed to bortezomib suffer from ER stress and initiate the unfolded protein response (UPR), a cellular program consisting of three branches that begin with ER overload sensor proteins ATF6, IRE1α and PERK. ATF6, itself a transcription factor, and XBP1, the downstream transcriptional effector of IRE1α, mainly activate the transcription of genes encoding ER chaperones and enzymes to reduce the ER protein processing burden. The initiator of the third UPR branch, PERK, phosphorylates eIF2α, thereby reducing translation initiation and triggering the integrated stress response (ISR) transcriptional program controlled by ATF4. Bortezomib additionally evokes ISR by activating another eIF2α kinase, HRI [5]. In contrast to most mRNAs, the phosphorylation of eIF2α increases the translation of the mRNA encoding ATF4 [6,7]. The genes activated by ATF4 include those that facilitate adaptation to stress as well as cell death-promoting genes, and the activity of the eIF2α–ATF4 pathway is regulated by several signaling mechanisms, including negative feedback regulation by the pseudokinase TRIB3.

In the present article, we characterize the effect of pharmacological modulators of the eIF2α–ATF4 pathway, and of TRIB3, an endogenous inhibitor of ATF4, on the gene expression and viability of HepG2 human hepatoma cells exposed to bortezomib. We show that pharmacological over-activation of the eIF2α–ATF4 pathway activity results in the elevation of all three UPR branches and sensitizes cells to bortezomib toxicity. In accordance, the suppression of eIF2α–ATF4, either pharmacologically or by TRIB3, increases the survival of bortezomib-treated cells. Taken together, the results indicate that TRIB3, a gene highly expressed in hepatic cancers, functions as a factor promoting resistance to bortezomib in hepatoma cells.

## 2. Results

### 2.1. Pharmacological Perturbation of the eIF2α–ATF4 UPR Branch Affects HepG2 Cell Sensitivity to Bortezomib

**Figure 1:**
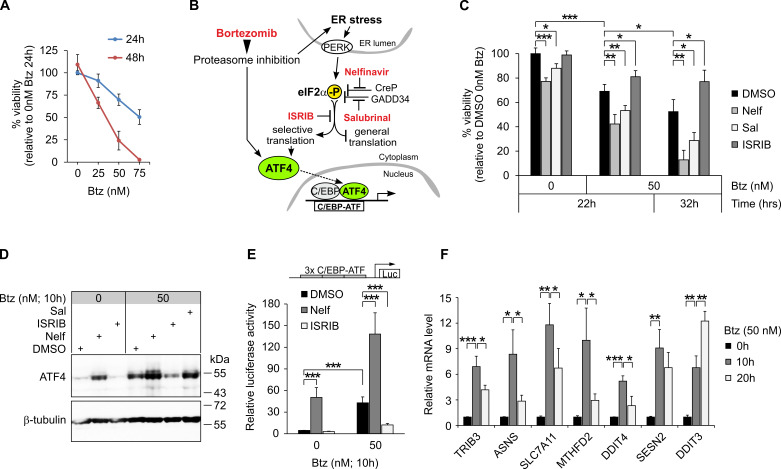
Pharmacological compounds targeting the eIF2α–ATF4 pathway affect bortezomib cytotoxicity in HepG2 cells. (**A**) Viability of HepG2 cells treated with different concentrations of bortezomib (Btz) for 24 or 48 h. Cell viability was measured using the Alamar Blue assay (*n* = 3–7) and is presented relative to vehicle-treated 24 h cells. (**B**) Schematic representation of the eIF2α–ATF4 stress response pathway in relation to Btz. Chemical modulators used in this study are highlighted in red. (**C**) Viability of HepG2 cells treated with Btz (50 nM), nelfinavir (Nelf; 20 µM), salubrinal (Sal; 20 µM), ISRIB (5 nM) or vehicle (DMSO), as indicated. Viability was measured using the Alamar Blue assay (*n* = 4–5) and is presented relative to DMSO-treated cells without Btz. (**D**) ATF4 protein immunoblot from HepG2 cells treated with Btz along with 20 µM Nelf, 20 µM Sal, 50 nM ISRIB or DMSO, as indicated. The blot shown is representative of two independent experiments. (**E**) Luciferase reporter assay for ATF4 transcriptional activity in cells treated as in panel D. Activity of the reporter plasmid containing three copies of the C/EBP–ATF composite site is presented relative to DMSO-treated (0 nM Btz) cells (*n* = 3–7). (**F**) RT-qPCR quantification of known ATF4 pathway target genes in HepG2 cells treated with Btz (*n* = 4). Data are presented relative to the average expression level in cells without Btz (0 h). For all panels, graphs display the means ± SD. * *p* < 0.05, ** *p* < 0.01, *** *p* < 0.001 using two-tailed *t* tests followed by Holm–Bonferroni correction.

Cancer cells exposed to proteasome inhibitors are known to activate the unfolded protein response, which can facilitate proteostasis (promoting cell survival), or, if cellular adaptations prove insufficient, give way to ER stress-induced cell death [3]. To investigate the net effect of eIF2α–ATF4 pathway activity on hepatoma cell susceptibility to proteasome inhibition, we first determined the concentration of bortezomib to induce ~50% cell death after 24 h (Figure 1A) and then combined bortezomib treatment with pharmacological compounds that specifically augment or neutralize eIF2α phosphorylation [8] (Figure 1B). As shown in Figure 1C, 50 nM bortezomib led to approximately 30% and 50% decreased cell viability after 22 and 32 h treatment, respectively. Co-treatment of the cells with ISRIB, a compound that renders cells insensitive to phospho-eIF2α [9], resulted in a significant delay in bortezomib-induced cell death (Figure 1C). Nelfinavir or salubrinal, compounds which cause cells to accumulate phospho-eIF2α by inhibiting eIF2α phosphatase cofactors CReP and GADD34, respectively [10,11], demonstrated some toxicity towards HepG2 cells already as single treatments, whereas in combination with bortezomib, dramatically reduced cell viability was seen (Figure 1C).

To evaluate whether these treatments produced the expected molecular outcomes, we analyzed the protein levels of ATF4, the key transcriptional effector of the eIF2α pathway [8] and a gene directly coupled at the translational level to eIF2α phosphorylation status [6,7]. As depicted in Figure 1D, the ATF4 level was markedly induced by bortezomib and nelfinavir, and was further augmented by the combination of bortezomib and nelfinavir. ISRIB co-treatment on the other hand, as expected, blunted ATF4 increase in response to bortezomib (Figure 1D). To measure the functional output of the ATF4 protein, we employed a luciferase reporter construct carrying three copies of a C/EBP–ATF composite site [12], the principal binding motif for ATF4 [8]. The results of the reporter assay, shown in Figure 1E, revealed that ATF4 transcriptional activity dynamics follow the overall ATF4 protein levels (Figure 1D), demonstrating the expected positive responses to bortezomib, nelfinavir and their combination, and a decrease in ATF4 activity when ISRIB is present along with bortezomib. RT-qPCR assessment of several ATF4-dependent genes [13,14] revealed robust upregulation in bortezomib-treated cells but with differences in the timing of the maximal observed expression level (Figure 1F), possibly indicating feedback regulator actuation [15,16]. Taken together, the results indicate strong but finely tuned activation of ATF4 in bortezomib-treated HepG2 cells and the potential to affect cell sensitivity to bortezomib by modulating the activity of the eIF2α–ATF4 pathway.

### 2.2. Transcriptional Profiling of Hepatoma Cells Treated with Bortezomib in Combination with Nelfinavir or ISRIB

**Figure 2:**
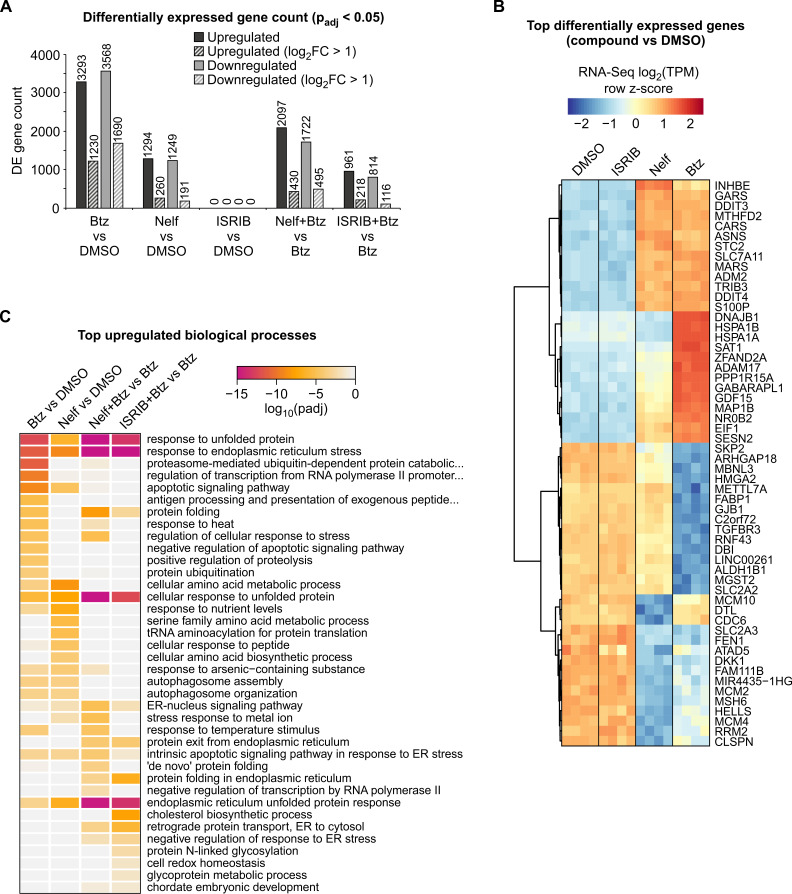
RNA-Seq profiling of bortezomib (Btz) and compounds targeting eIF2α–ATF4 signaling. HepG2 cells were treated for 10 h with Btz (50 nM), nelfinavir (Nelf; 20 µM), ISRIB (50 nM) or vehicle (DMSO), as indicated (*n* = 4). (**A**) Differentially expressed (DE) gene counts for the indicated pairwise comparisons of gene expression profiles. (**B**) Heatmap depicting the expression levels of most significantly up- and downregulated genes for Btz and Nelf monotreatments. No genes were significantly regulated by ISRIB monotreatment (panel **A**). Each column represents an independent library. (**C**) Gene ontology enrichment profiling for the effect of single-compound treatments and the effect of co-treatment with Nelf or ISRIB on the response to Btz. The GO Biological Process database was used, the most significant categories for each query gene list were selected, and the resulting categories were plotted for all the gene expression comparisons.

To obtain a global view of the transcriptional responses induced in bortezomib-treated HepG2 cells, with or without nelfinavir and ISRIB, we carried out RNA-Seq gene expression profiling. The results revealed that 10 h of bortezomib treatment causes extensive changes in gene expression, with >1000 genes up- and downregulated >2-fold (Figure 2A and Table S1). As expected, there are several protein chaperons among the most strongly upregulated genes (Figure 2B) and functional profiling revealed the responses to unfolded protein and ER stress as the most significantly enriched biological processes for bortezomib-upregulated genes (Figure 2C). Top downregulated genes in the response to bortezomib contained multiple cell division genes of the MCM gene group (Figure 2B), indicating a cessation of the cell cycle.

Nelfinavir, in the absence of bortezomib, regulated several hundred genes >2-fold (Figure 2A and Table S1). Supporting its expected molecular effect of ATF4 induction, the most highly nelfinavir-upregulated genes in HepG2 cells were almost all previously characterized ATF4 direct targets (Figure 2B) [13]. Functional profiling indicated that nelfinavir activated the ER stress response/UPR, and, characteristically, processes related to amino acid biosynthesis and tRNA loading (Figure 2C), a group of known ATF4 target genes [13,14]. ISRIB, in the absence of bortezomib, did not result in any significantly regulated genes (Figure 2A and Table S1), which may be explained by its biochemical mechanism of action and the expected lack of phosphorylated eIF2α in unstressed cells [17].

**Figure 3:**
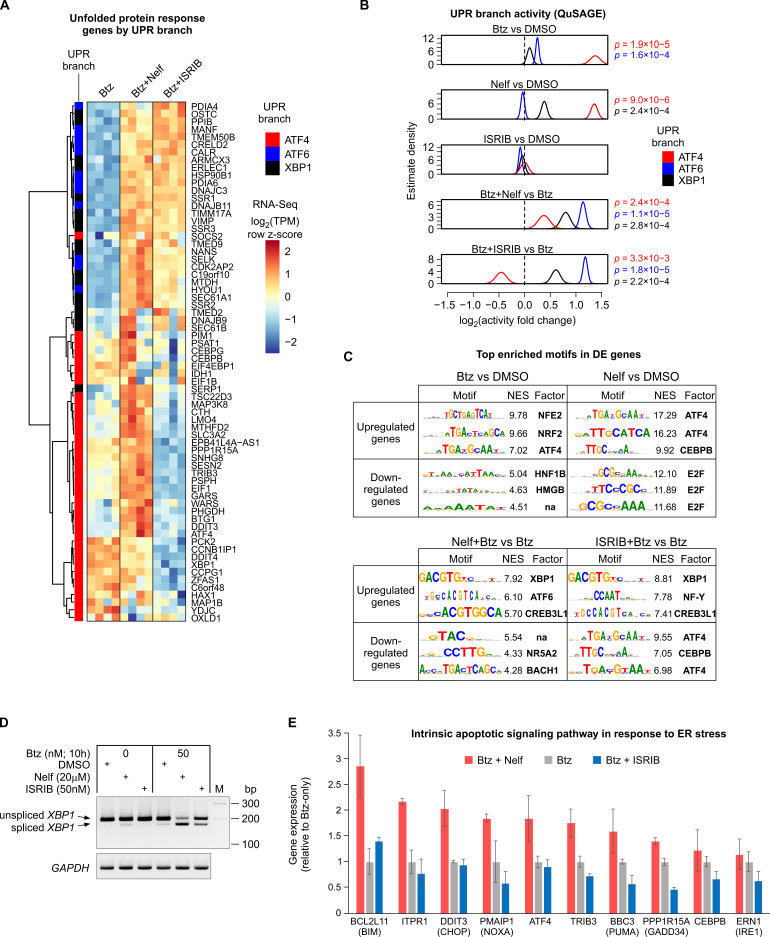
Perturbation of eIF2α–ATF4 signaling alters the activity of all three branches of the UPR and the expression of pro-apoptotic ER stress response genes. RNA-Seq data from HepG2 cells treated with bortezomib (Btz), nelfinavir (Nelf) and ISRIB (Figure 2). (**A**) Alteration of the Btz response by co-treatment with Nelf or ISRIB. Genes previously assigned to one of the three UPR branches (ATF4, ATF6 or XBP1) were included in the heatmap. Each column corresponds to an independent library. (**B**) The calculated activity of individual branches of the UPR at the gene set level. Genes were assigned to UPR branches as in panel A and the combined expression change was calculated using the QuSAGE method. (**C**) Transcription factor motif over-representation analysis of the effect of Nelf and ISRIB co-treatment on the Btz response. Up- or downregulated genes were subjected to genomic motif enrichment analysis using i-cisTarget. (**D**) RT-PCR quantification of spliced and unspliced *XBP1* mRNA in HepG2 cells. The agarose gel image is representative of two independent experiments. (**E**) RNA-Seq gene expression levels (means ± SD; *n* = 4) for genes of the intrinsic apoptotic signaling pathway in response to ER stress. The gene expressions are presented relative to Btz monotreatment.

When applied in combination with bortezomib, nelfinavir and ISRIB affected the expression of several hundred genes compared to bortezomib alone, indicating an altered response to bortezomib (Figure 2A and Table S1). Previous studies have reported that the expression level of proteasome complex genes is a determinant of proteasome inhibitor sensitivity [18,19,20,21,22]. In our RNA-Seq experiments, bortezomib treatment resulted in a strong upregulation of proteasome complex genes; however, this gene induction does not appear to be altered by co-treatment with nelfinavir or ISRIB (Figure S1A,B). Rather, gene enrichment profiling of the upregulated genes indicated primarily that ER stress processes are being overactivated by bortezomib co-treatment with nelfinavir or ISRIB (Figure 2C). Since the overall ER stress response is composed of three ‘branches’, three sensor proteins coupled to specific transcription factors (ATF4, ATF6 and XBP1) [23], we set out to assess the activity of the UPR branches individually. Adamson et al. (2016) [24] was able to assign subsets of UPR target genes to the specific transcription factors. Plotting these gene sets from bortezomib-treated cells (with or without nelfinavir and ISRIB co-treatment) revealed that, as expected, nelfinavir co-treatment resulted in over-activation of the ATF4 branch genes and, conversely, ISRIB co-treatment inhibited the ATF4 branch of UPR genes (Figure 3A). Additionally, supplementing bortezomib with either ISRIB or nelfinavir appeared to generate an overactivation of the XBP1 and ATF6 branches of the UPR (Figure 3A). To quantify this observation, we applied QuSAGE analysis, a method to evaluate fold change for entire sets of genes [25]. The results confirmed that supplementing bortezomib with either nelfinavir or ISRIB leads to significant upregulation of the ATF6 and XBP1 branches of the UPR, while having the expected effects on the ATF4 pathway (activation by nelfinavir; inhibition by ISRIB) (Figure 3B). To evaluate these effects independently of the Adamson et al. [24] UPR branch gene sets, we carried out transcription factor motif enrichment analysis for genes differentially expressed between bortezomib monotreatment and bortezomib combined with nelfinavir or ISRIB. The results revealed that genes upregulated by nelfinavir alone displayed a very strong enrichment for the ATF4 motif, as do ISRIB-downregulated genes (Figure 3C). Furthermore, the motif for XBP1 was the most highly enriched motif among genes upregulated by bortezomib co-treatment with nelfinavir or ISRIB, compared to bortezomib alone (Figure 3C). The active form of XBP1 protein is produced following the excision of a short intron from its mRNA [26]. PCR assessment of the ratio of unspliced to spliced XBP1 transcripts revealed that bortezomib alone induced mild but detectable splicing of the XBP1 mRNA, and the proportion of spliced XBP1 transcript was elevated by nelfinavir or ISRIB co-treatment (Figure 3D). Taken together, the results confirm that nelfinavir and ISRIB are potent in activating and inhibiting, respectively, the ATF4 pathway, and when applied in combination with bortezomib, generate an overactivation of the ATF6 and XBP1 branches of the ER stress response.

As shown in Figure 2C, the combination of bortezomib and ISRIB, compared to bortezomib alone or in combination with nelfinavir, led to decreased enrichment of ER stress response intrinsic apoptotic signaling pathway genes. Querying the RNA-Seq data for genes of this ontology category revealed a set of genes, including *BCL2L11* (BIM), *ITPR1*, *DDIT3* (CHOP), *PMAIP1* (NOXA), *ATF4*, *TRIB3*, *BBC3* (PUMA), *PPP1R15A* (GADD34), *CEBPB* and *ERN1* (IRE1) (Figure 3E), that demonstrate bortezomib-induced upregulation, which is blunted by ISRIB co-treatment or augmented by nelfinavir co-treatment. Thus, these genes represent candidate mediators for bortezomib-induced cell death and the effect of eIF2α–ATF4 pathway modulating compounds on the cell death process.

### 2.3. The ATF4 Regulatory Factor TRIB3 Is Highly Expressed in Hepatoma Cells and Correlates with Resistance to Bortezomib

**Figure 4:**
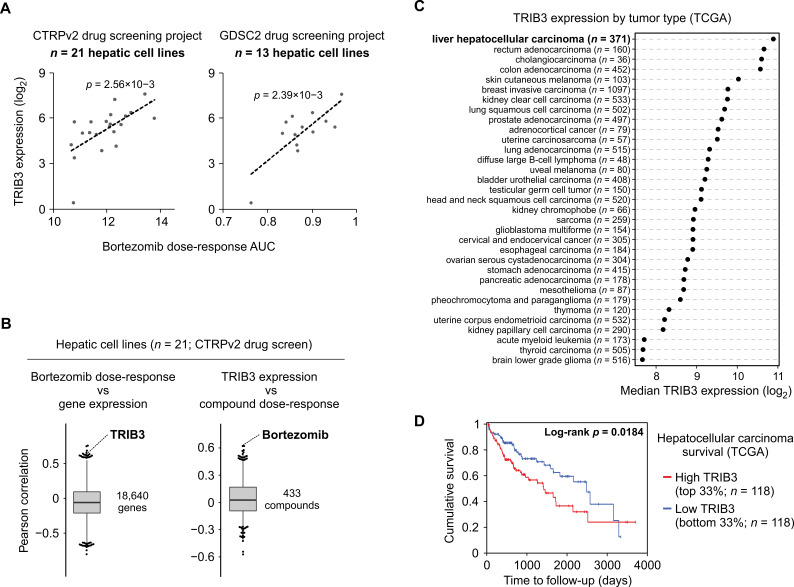
The expression level of TRIB3 is high in liver tumors and correlates with resistance to bortezomib in hepatic cancer cell lines. (**A**) Bortezomib sensitivity data from two drug screening projects combined with the RNA-Seq gene expression data of the liver cancer cell lines studied. The linear regression p value is shown. (**B**) Reciprocal high ranking of the relationship between *TRIB3* gene expression level and bortezomib dose-response in hepatic cancer cell lines. Correlation was calculated between bortezomib dose-response and all expressed genes (left panel), or between *TRIB3* gene expression and all studied compounds (right panel). (**C**) *TRIB3* gene expression level by tumor type. The Cancer Genome Atlas (TCGA) RNA-Seq data from primary tumor samples was used. (**D**) High *TRIB3* expression is associated with poor survival in hepatocellular carcinoma patients. TCGA samples were stratified by their *TRIB3* mRNA level (top and bottom 33%) and subjected to survival analysis.

To investigate the mechanisms of bortezomib sensitivity in human hepatoma cells, we combined previously published dose-response data from two drug screening projects (CTRPv2 and GDSC2 [27,28]) with the latest RNA-Seq-based gene expression data for human cell lines (CCLE [29]). Among hepatoma cell lines, cell viability data from both drug screening projects revealed a positive correlation between resistance to bortezomib and the expression level of *TRIB3* (Figure 4A), a gene upregulated by bortezomib (Figure 1F and 2B) and known to be a negative regulator of the ATF4 pathway [12,15,30]. In the CTRPv2 data, which features 21 hepatic cell lines, the relationship between *TRIB3* and bortezomib is highly reciprocal: *TRIB3* ranks 8th highest out of all genes for correlation with resistance to bortezomib, and bortezomib ranks 2nd highest out of all compounds for compound resistance correlation with *TRIB3* expression (Figure 4B). To assess the expression levels of *TRIB3* in liver tumors in vivo, we ranked the tumor types from TCGA [31] by median expression of *TRIB3*, which revealed that hepatocellular carcinoma has the highest median *TRIB3* level out of all TCGA tumor types (Figure 4C). Furthermore, stratifying the hepatocellular carcinoma cases of the TCGA cohort by their *TRIB3* expression level revealed that a higher level of *TRIB3* is associated with poorer survival (Figure 4D). Since pharmacological perturbation of ATF4 activity affected cell sensitivity to bortezomib (Figure 1C), these results raise the possibility that hepatoma cells employ high *TRIB3* expression to achieve optimal ATF4 pathway activity and thereby survival under stressful conditions.

### 2.4. Generation and Characterization of HepG2 Cells Expressing Endogenous TRIB3 Fused with Flag-Tag (HepG2-TRIB3-Flag Cells)

Studies into the protein–protein interaction between ATF4 and TRIB3 revealed that TRIB3 binds to the transactivation domain of ATF4, leaving the DNA binding ability of ATF4 intact [30]. Moreover, TRIB3 is reported to also bind other transcription factors, such as CHOP (DDIT3), p65/NF-κB, C/EBPβ and PPARγ [32,33,34,35]. However, at a genome-wide level, the association of TRIB3 to chromatin has not been studied, leaving open questions of preferred binding partners, motifs and loci.

**Figure 5:**
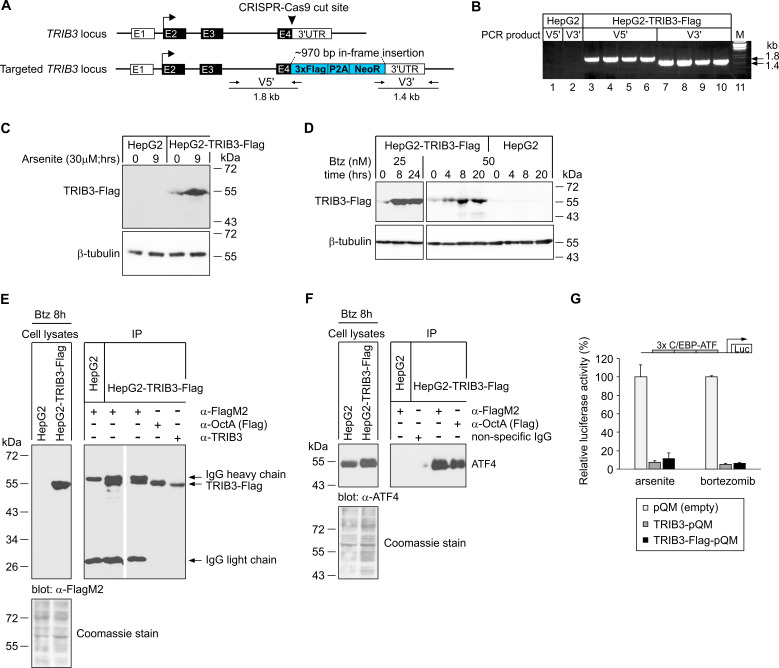
Generation and characterization of HepG2 cells expressing endogenous TRIB3 fused with Flag tag (HepG2-TRIB3-Flag cells). (**A**) Schematic representation of CRISPR-directed recombination in the *TRIB3* locus, resulting in TRIB3 protein fusion with Flag tag. Boxes E1–E4 denote *TRIB3* exons (the coding region is in the black background) and the translational initiation codon is marked with an arrow in exon 2. At the bottom, small arrows denote PCR amplicons used to validate the integration of the Flag donor cassette from 5′ (V5′) and 3′ direction (V3′). (**B**) PCR validation of Flag donor cassette insertion into the *TRIB3* locus. Lanes 3 and 7 are from cell pools, while lanes 4–6 and 8–10 represent individual clones. (**C**,**D**) Stress-induced upregulation of endogenous TRIB3 fused with Flag tag. HepG2-TRIB3-Flag cells or unedited HepG2 cells were exposed to arsenite (**C**) or bortezomib (Btz; **D**). Anti-Flag antibody was used for immunoblotting, along with anti-β-tubulin as a loading control. The blots shown are representative of 4 (**C**) or 2 (**D**) experiments. (**E**) Immunoprecipitation (IP)-based confirmation of TRIB3-Flag protein identity. Monoclonal M2 anti-Flag, polyclonal OctA-Probe anti-Flag and anti-TRIB3 antibodies were used for immunoprecipitation, and monoclonal M2 anti-Flag antibody was used for detection. (**F**) ATF4 coimmunoprecipitates with TRIB3-Flag. Proteins were immunoprecipitated from cell lysates using monoclonal M2 anti-Flag, polyclonal OctA-Probe anti-Flag or non-targeting IgG. Anti-ATF4 antibody was used for detection. (**G**) The C-terminally fused 3xFlag tag does not affect the ability of TRIB3 to inhibit the transcriptional activity of ATF4. HepG2 cells were cotransfected with human *TRIB3* promoter reporter construct containing three C/EBP–ATF sites and expression plasmid for either TRIB3 (TRIB3-pQM), TRIB3 fused with Flag tag (TRIB3-Flag-pQM) or empty vector (pQM). The cells were exposed to 30 µM arsenite or 50 nM Btz for 9 h. The reporter activities (mean ± SD) from two independent experiments are shown normalized to the reporter activity in control (pQM-transfected) cells.

To localize chromatin binding sites of TRIB3 genome-wide, we decided to harness CRISPR epitope tagging ChIP-seq (CETCh-seq) [36], which aims to fuse an epitope tag to the C-terminus of the endogenous protein, preserving the native regulation of the gene. HepG2 were transfected with plasmid encoding CRISPR gRNA and Cas9 nuclease along with the pFETCh homologous recombination donor plasmid containing the Flag cassette (Figure 5A) and homology arms for targeting *TRIB3* C-terminus. Cells with successful targeting are expected to produce TRIB3 protein fused with 3xFlag epitope and neomycin resistance protein, separated by a P2A self-cleaving peptide.

As shown in Figure 5B, PCR products validating the homologous recombination event were evident from the edited cells. Immunoblot analysis revealed that in the G418-resistant HepG2-TRIB3-Flag cells, anti-Flag antibody recognizes a protein that is absent in the parental HepG2 cells and has the expected mobility of TRIB3 fused with 3xFlag (Figure 5C). Importantly, responsiveness to cellular stress has been preserved in the targeted *TRIB3* locus: exposure to bortezomib strongly upregulated TRIB3-Flag (Figure 5D), as did treatment with the oxidative stress agent arsenite (Figure 5C), a known inducer of TRIB3 [37]. This protein can be immunoprecipitated by anti-TRIB3 antibody, monoclonal M2 anti-Flag antibody and polyclonal OctA-Probe anti-Flag antibody (Figure 5E), further confirming it as the expected TRIB3-Flag fusion.

To verify that the C-terminally fused 3xFlag epitope does not affect the ability of TRIB3 to interact with ATF4, we confirmed that ATF4 co-immunoprecipitates with TRIB3-Flag in experiments performed with monoclonal M2 anti-Flag antibody as well as with polyclonal OctA-Probe anti-Flag antibody (Figure 5F). To show that the interaction between ATF4 and TRIB3-Flag has the same functional outcome as with untagged TRIB3, luciferase reporter assays for ATF4 transcriptional activity were carried out using a human *TRIB3* promoter construct containing three C/EBP-ATF composite sites which are responsive to ATF4 [12]. The results, shown in Figure 5G, demonstrated that the Flag-tagged and untagged TRIB3 are similarly potent in inhibiting ATF4-driven reporter gene expression in cells exposed to arsenite or bortezomib.

### 2.5. TRIB3 Co-Localizes with ATF4 on Chromatin Genome-Wide

To elucidate the chromatin binding pattern of TRIB3, we used the HepG2-TRIB3-Flag cells to carry out ChIP-Seq of TRIB3 in untreated and bortezomib-treated cells. To complement this data, we performed ChIP-Seq for ATF4 upon bortezomib treatment.

**Figure 6:**
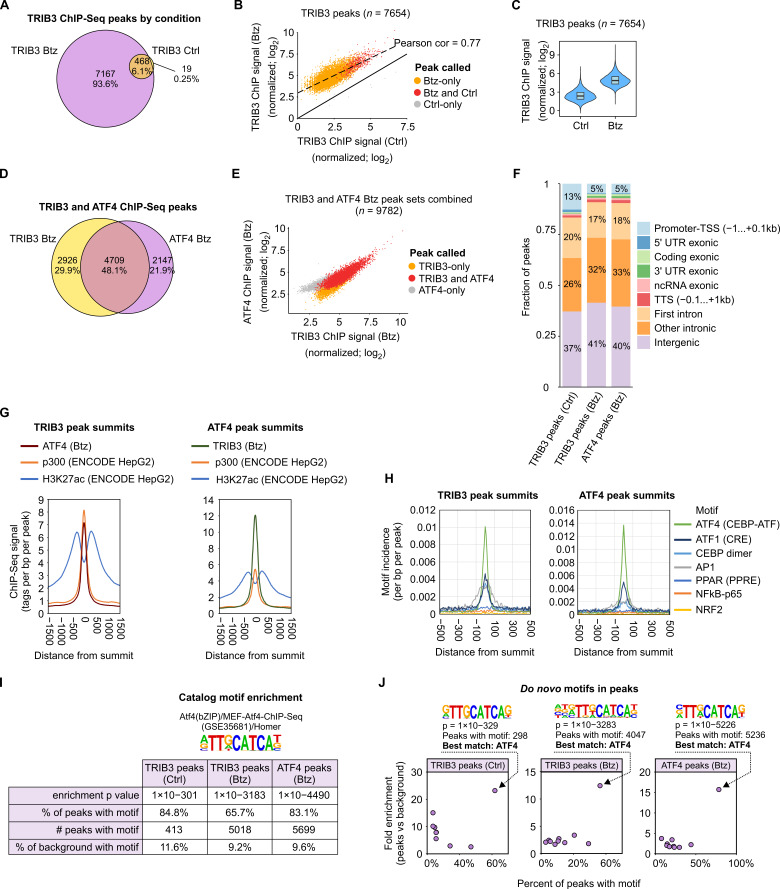
TRIB3 localization to chromatin is highly concordant with the binding pattern of ATF4. For ChIP-Seq, HepG2-TRIB3-Flag cells were treated with 50 nM bortezomib (Btz) for 9 h or mock treated (Ctrl), and chromatin was immunoprecipitated using anti-Flag or anti-ATF4 antibody. (**A**) Number of TRIB3 chromatin peaks detected per treatment condition. (**B**) TRIB3 ChIP-Seq signal strength at regions detected as TRIB3 peaks either in Ctrl, Btz or both conditions. (**C**) Violin plot of the TRIB3 ChIP-Seq signal strength across all peaks in Ctrl and Btz conditions. (**D**) Overlap of peak regions detected in TRIB3 and ATF4 ChIP-Seq-s in Btz conditions. (**E**) Correlation between TRIB3 and ATF4 ChIP-Seq signal strength in peak regions detected for one or both proteins. (**F**) The genomic context of TRIB3 and ATF4 ChIP-Seq peaks. (**G**) ChIP-Seq signal colocalization histograms centered on the peak summits of TRIB3 or ATF4 peaks. The ChIP-Seq data for coactivator p300 and histone mark H3K27ac were obtained from ENCODE for HepG2 cells. (**H**) Motif incidence histograms centered on the peak summits of TRIB3 or ATF4 peaks. (**I**) Detection statistics of a previously defined ATF4 binding motif. (**J**) Results of de novo motif finding using HOMER. Fold enrichment is the ratio between the motif incidence in the peak set and the motif incidence in the automatically selected background region set.

The data revealed that TRIB3 is extensively associated to chromatin, with >7000 peaks in the consensus peak set (Figure 6A and Table S2). The number of TRIB3 peaks detected in bortezomib-treated cells was >10-fold higher than in control cells (Figure 6A), in line with the increased expression level of TRIB3 protein (Figure 5D). Nearly all TRIB3 peaks in control cells were also evident in bortezomib-treated cells (Figure 6A), and the TRIB3 peaks detected in untreated cells were among the strongest peaks in bortezomib-treated cells (Figure 6B), suggesting similar binding modes in the stressed and unstressed conditions. Indeed, when comparing the TRIB3 ChIP-Seq peak signals between bortezomib-treated and control cells, there was a mostly uniform increase in TRIB3 chromatin association across all peak regions in response to bortezomib (Figure 6C).

ChIP-Seq of ATF4 in bortezomib-treated cells identified nearly 7000 peaks, of which 4700 were also bound by TRIB3 (Figure 6D and Table S2), demonstrating a large and reciprocal co-localization of ATF4 and TRIB3 on chromatin. Furthermore, analyzing ChIP-Seq signal strength across the union of TRIB3 and ATF4 peaks revealed that ChIP-Seq signals for TRIB3 and ATF4 were strongly and positively correlated across the binding loci (Figure 6E).

The genomic contexts of TRIB3 and ATF4 peaks were highly similar, with most peaks located outside of proximal promoter regions, in either intergenic or intronic regions (Figure 6F). This fits the notion that ATF4 does not require a fixed position relative to the transcription start site (TSS) [38,39,40]. To assess the chromatin state context at TRIB3 and ATF4 binding sites, we utilized ENCODE project ChIP-Seq data for the H3K27ac histone mark and the p300 histone acetyltransferase from (untreated) HepG2 cells [41]. The ChIP signal histograms, centered on the summits of either TRIB3 or ATF4 peaks (Figure 6G), demonstrated that TRIB3 and ATF4 co-localized extensively with H3K27ac, a mark of active enhancers and promoters [42], as well as with p300, a transcription co-activator thought to be recruited by ATF4 [43]. Furthermore, ATF4 and TRIB3 showed a strong concentric binding pattern with each other (Figure 6G).

Averaged across a large set of ChIP peaks, the DNA motif(s) directly responsible for the ChIP signal is expected to be enriched most strongly at the peak summit. Based on reported protein–protein interaction partners for TRIB3 [32,33,34,35,44] and binding motifs/partners for ATF4 [13,45], we selected motifs that could potentially mediate their DNA binding. Motif incidence histograms, centered on TRIB3 and ATF4 peak summits, showed a steep incidence peak for the C/EBP–ATF motif at the summits (Figure 6H), implicating direct interaction with this motif. Other candidates for directly bound motifs were CRE/ATF1 and C/EBP dimer motifs, while AP1 motif enrichment was more diffuse, indicating a cooperative mode of interaction (Figure 6H). No enrichment was evident for PPAR, NF-κB and NRF2 motifs (Figure 6H).

We further subjected the peak sequences to motif enrichment analysis for all motifs in the HOMER known motif catalog as well as de novo motif finding. The analysis of catalog motifs in TRIB3 or ATF4 ChIP-Seq peaks revealed the presence of the ATF4 motif (specifically, the C/EBP–ATF motif) as present in 66–85% of peaks and highly enriched over background regions (Figure 6I and Table S3). Similarly, for both ATF4 and TRIB3 binding regions, motifs corresponding to the C/EBP–ATF motif stood out as the only de novo motifs found, demonstrating high enrichment over background and high fraction of peaks with a motif (Figure 6J and Table S3). Thus, the primary DNA motif mediating TRIB3 (and ATF4) association to chromatin appears to be the C/EBP–ATF motif where the binding patterns of ATF4 and TRIB3 correlate extensively, suggesting ATF4 as the primary mediator of TRIB3 association to chromatin.

### 2.6. Enrichment of TRIB3 Binding Near Bortezomib-Upregulated Genes Involved in the ER Stress Response and Cell Death

**Figure 7:**
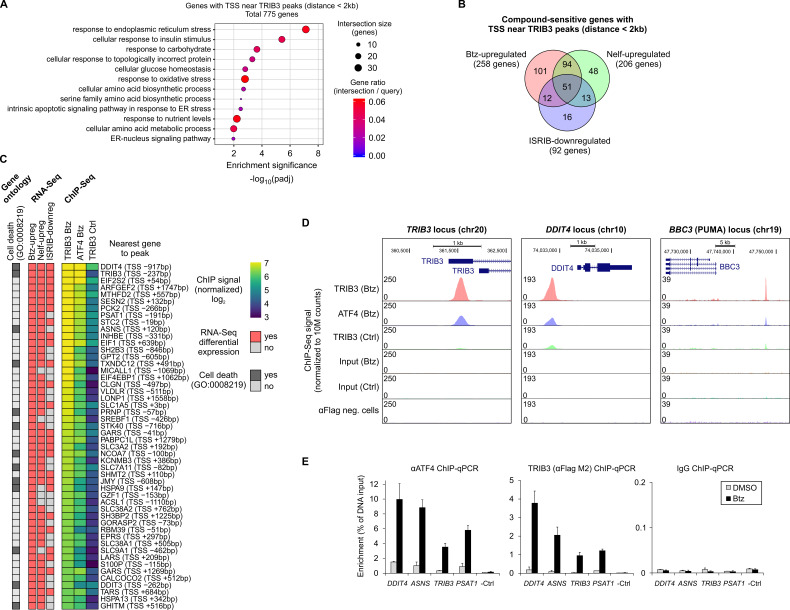
TRIB3 and ATF4 binding near genes that are regulated in response to bortezomib (Btz), nelfinavir (Nelf) and ISRIB in HepG2 cells. (**A**) Gene ontology enrichment analysis for genes with a TRIB3 ChIP-Seq peak within 2 kb of the transcription start site (TSS). The GO Biological Process database was used. (**B**) RNA-Seq differential expression results overlap with genes harboring TRIB3 ChIP-Seq peaks (TSS ± 2 kb). ISRIB-downregulated genes were obtained comparing ISRIB and Btz co-treatment to Btz alone. (**C**) Btz-upregulated genes with TSS-proximal (TSS ± 2 kb) TRIB3 ChIP-Seq peaks. Genes are ranked by the TRIB3 Btz ChIP-Seq signal strength. (**D**) ChIP-Seq coverage plots for selected loci. The αFlag neg. cells track represents ChIP-Seq of unedited HepG2 cells with anti-Flag antibody. (**E**) ChIP-qPCR validation for the binding of ATF4, TRIB3 (anti-Flag M2) and non-targeting IgG at selected loci detected in ChIP-Seq. A negative control locus is also shown (-Ctrl). The means ± SD of 2–5 ChIP experiments from two biological replicates are shown. The DNA quantity is presented as a percentage of input chromatin.

To predict which biological processes and genes could be affected by TRIB3 binding to chromatin, we annotated the peaks in the TRIB3 consensus peak set (Figure 6A) to their nearest gene and retained only cases where the distance to the TSS was <2 kb, resulting in a list of 775 genes (Table S2). Gene ontology over-representation analysis of this ChIP-Seq-derived gene set revealed the most significant enrichment for ER stress response, related categories such as response to topologically incorrect protein, and intrinsic apoptotic signaling (Figure 7A), which were also uncovered in functional profiling of the RNA-Seq results for bortezomib-upregulated genes (Figure 2C). Specifically, out of the 775 genes that have their TSS within 2kb of a TRIB3 ChIP-Seq peak, 258 were bortezomib-upregulated genes based on RNA-Seq (Figure 7B), thus representing genes within the bortezomib response that are possibly sensitive to TRIB3. There was also a considerable overlap between the 775 TRIB3 ChIP-Seq genes and the putatively ATF4-regulated genes, i.e., the nelfinavir-upregulated and ISRIB-downregulated gene sets (Figure 7B).

Figure 7C depicts the strongest TRIB3 ChIP-Seq peaks that are in the vicinity of bortezomib-upregulated genes. The results showed that the promoter of TRIB3 itself is among the most abundantly bound loci by TRIB3 (Figure 7C,D), with many other well-known cellular stress response genes and ATF4 target genes also ranking highly [8,13,14]. Notably, multiple genes assigned to the cell death process by gene ontology displayed TRIB3 association to chromatin near their promoters (Figure 7C), including the strongest TRIB3 binding signal detected in this analysis, the *DDIT4* promoter (Figure 7D). Furthermore, these TSS-proximal TRIB3 binding loci (Figure 7C) demonstrated increased TRIB3 binding signal in bortezomib-treated cells compared to untreated cells, a trend confirmed in ChIP-qPCR validation experiments using a subset of regions (Figure 7E). Beyond TSS-proximal regions, a promising TRIB3 binding region is approximately 12 kb upstream of *BBC3* (Figure 7D), which encodes the pro-apoptotic protein PUMA and displays an ISRIB and nelfinavir sensitive expression pattern (Figure 3E). Taken together, TRIB3 binding regions occur near a considerable number of bortezomib-upregulated genes and their occupancy by TRIB3 increases upon bortezomib stress.

### 2.7. TRIB3 Controls ATF4 to Maintain Balanced Activity of the UPR Branches and Cell Viability upon Exposure to Bortezomib

**Figure 8:**
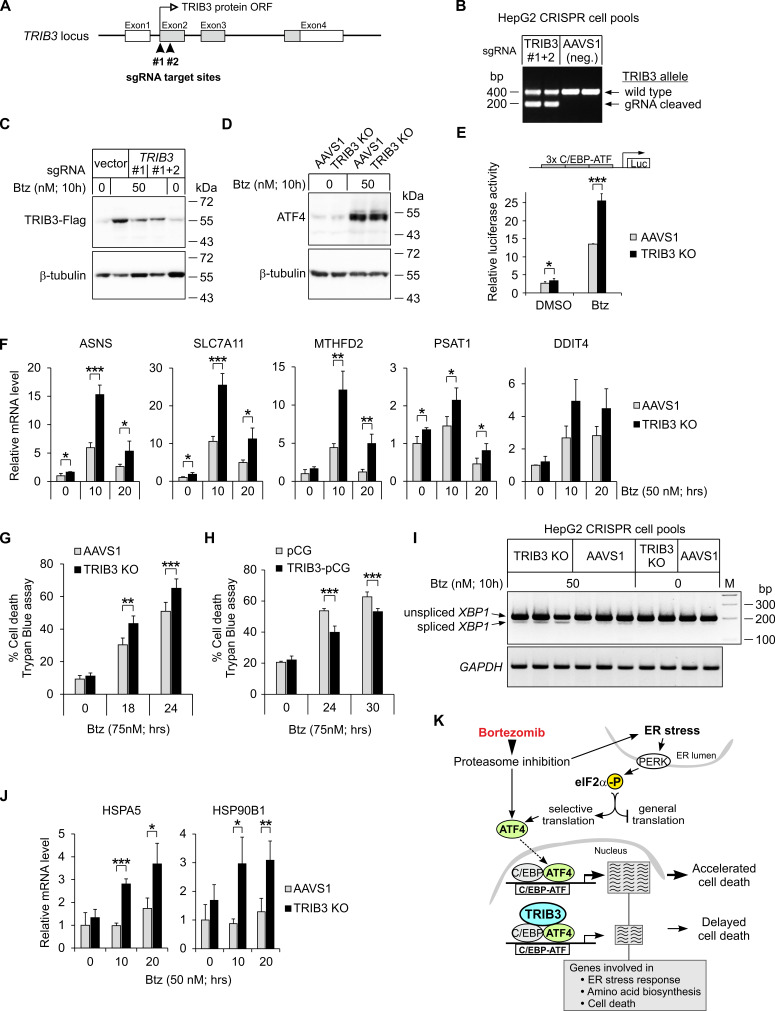
TRIB3 reduces ATF4 transactivation activity, lowers ER stress response levels and promotes survival of HepG2 cells exposed to bortezomib (Btz). (**A**) Schematic illustration of CRISPR/Cas9-based disruption on the human *TRIB3* gene. Guide RNA target sites (#1; #2) are indicated by filled arrowheads. (**B**) PCR conformation of targeted deletion in the *TRIB3* locus. Agarose gel of PCR products from HepG2 cell pools transfected with *TRIB3*-specific sgRNAs (#1 and #2) or with control sgRNA targeting the AAVS1 locus (two representative transfections out four are shown). (**C**) Immunoblot validation of *TRIB3* gene knockout in HepG2-TRIB3-Flag cells treated with Btz. The anti-Flag M2 antibody was used to detect TRIB3-Flag. (**D**) ATF4 protein levels in Btz-treated HepG2 *TRIB3* knockout (KO) and control (AAVS1-targeted) cells. Immunoblot analysis with anti-ATF4 antibody is representative of three experiments. (**E**) ATF4 transcriptional activity in *TRIB3* knockout and control (AAVS1-targeted) HepG2 cells, as measured by the luciferase assay using a reporter plasmid containing three copies of C/EBP–ATF motif (means ± SD from *n* = 4). (**F**,**J**) RT-qPCR quantification of ATF4 target genes (**F**) and ER chaperon genes (**J**) in Btz-treated HepG2 *TRIB3* knockout and control (AAVS1-targeted cells) (*n* = 4). Data are presented relative to the average expression level in AAVS1-targeted cells without Btz. (**G**,**H**) Effect of *TRIB3* disruption (**G**) or overexpression (**H**) on Btz-induced cell death. Total 4000–6000 (**G**) or 1000–3000 (**H**) cells were counted with Trypan Blue staining. The mean percentage ± SD of non-viable (Trypan-positive) cells is presented from 5–8 independent experiments. (**I**) Effect of *TRIB3* disruption on *XBP1* mRNA splicing. The agarose gel showing RT-PCR products is representative of four experiments. (**K**) Schematic summary of ATF4 and TRIB3 involvement in the bortezomib stress response. * *p* < 0.05, ** *p* < 0.01, *** *p* < 0.001 using two-tailed *t* tests followed by Holm–Bonferroni correction.

To shed light on the role of TRIB3 in the cells treated with bortezomib, we used CRISPR-Cas9 to disrupt the *TRIB3* gene. HepG2 cells were targeted by guide RNA-s at either the start of the *TRIB3* coding region (Figure 8A) or, as a control, at the AAVS1 safe harbor locus [46]. PCR-based assessment of *TRIB3* targeted deletion efficiency revealed that a significant fraction of *TRIB3* gene copies in cell pools transfected with *TRIB3*-specific sgRNAs have lost the DNA segment residing between the sgRNA cutting sites (Figure 8B). Immunoblotting confirmed that cells with CRISPR/Cas9-mediated disruption of the *TRIB3* gene display considerably reduced levels of TRIB3-Flag protein upon bortezomib treatment (Figure 8C).

Since TRIB3 localization to chromatin in bortezomib-treated cells is correlated with that of ATF4 (Figure 6E), we reasoned that reduced expression of TRIB3 might most directly affect the expression level of ATF4 target genes, disturbing the ATF4 branch of the UPR, and through this potentially leading to dysregulation of the other branches of UPR in the bortezomib-exposed cells, as was observed for ATF4-modulating compounds (Figure 1C and Figure 3A,B). While *TRIB3* disruption did not influence the ATF4 protein level in cells treated with bortezomib (Figure 8D), it led to a significant increase in transcriptional activity from a C/EBP–ATF response element-driven reporter construct (Figure 8E), indicating a de-repression of ATF4 activity in the cells. In line with this, significant over-activations were evident in *TRIB3* knockout cells (Figure 8F) for genes that demonstrate abundant presence of ATF4 and TRIB3 in their promoter regions by ChIP-Seq (Figure 7C).

Even though not all *TRIB3* gene copies in the cell pool were disrupted (Figure 8B,C), the TRIB3 knockout cell pools demonstrated increased cell death when subjected to bortezomib (Figure 8G). Conversely, plasmid-based overexpression of TRIB3 in unedited cells decreased cell death in response to bortezomib (Figure 8H). Neither TRIB3 disruption nor overexpression affected the viability of cells unexposed to bortezomib (Figure 8G,H). Similarly to cells with pharmacological overactivation of ATF4 (Figure 3D), cells with overactive ATF4 as a result of TRIB3 depletion also demonstrated enhanced splicing of XBP1 upon bortezomib exposure (Figure 8I) along with the induction ER stress markers that indicate an activation of XBP1 and ATF6 UPR branches, relative to control (AAVS1-targeted) cells treated with bortezomib (Figure 8J). Thus, TRIB3-mediated suppression of ATF4-dependent transcription at the chromatin level reduces bortezomib-induced cytotoxicity and enhances HepG2 cell survival (Figure 8K).

## 3. Discussion

Liver cancer is a major world-wide public health issue with an urgent need for new therapies and an improved understanding of the molecular mechanisms involved in malignant cell survival and treatment resistance. Previous studies have found that the proteasome inhibitor bortezomib triggers tumor cell death in hepatocellular carcinoma models in vitro and in vivo [19,47,48,49]; however, a phase II trial studying bortezomib as a single-agent treatment in patients with advanced, unresectable hepatocellular carcinoma revealed minimal clinical improvement [50]. In the current article, we study the transcriptional response programs and cell viability of hepatocellular carcinoma cells exposed to bortezomib in combination with pharmacological compounds that perturb intracellular signaling to increase (nelfinavir, salubrinal) or decrease (ISRIB) the level of ATF4, a key transcriptional regulator of cellular stress responses. Our data indicate that the intrinsic apoptotic signaling pathway in response to ER stress is activated when the cells are exposed to bortezomib along with compound increasing ATF4 levels, and that perturbing the activity of the ATF4 pathway leads to over-activation of the ATF6 and XBP1 pathways, the other two arms of the UPR. We further uncover that TRIB3, an ATF4 binding protein particularly highly expressed in liver tumors, associates to chromatin at most ATF4 binding sites, limiting the transcriptional output and inhibiting bortezomib-induced cell death.

Combining ChIP-Seq with epitope tagging of endogenous TRIB3 allowed for the first time a genome-wide view of genes which are potentially regulated by TRIB3 at the chromatin level. In bortezomib-exposed hepatoma cells, we found that TRIB3 resides predominantly at genomic regions containing the C/EBP–ATF motif, which we and others have previously described as the regulatory motif mediating the upregulation of a number of ATF4 target genes in response to cellular stress [12,13,45,51,52]. ATF4 binds to C/EBP–ATF sites as a heterodimer with C/EBP transcription factors and the binding sites can reside in promoters, introns or intergenic regions, and they have been shown to satisfy the positional and orientational definitions of enhancer elements [38,39,40]. To a much lesser extent than C/EBP–ATF motifs, we detected enrichment of other bZIP transcription factor motifs (ATF1/CRE, C/EBP dimer and AP1) in TRIB3 ChIP-Seq peaks. The ATF1/CRE motif is reported to bind ATF4 and the enrichment of this motif is also observed as a minor motif in ATF4 peaks; therefore, the binding of TRIB3 to chromatin at ATF1/CRE motif could also be mediated by ATF4. The enrichment of C/EBP dimer and AP1 motifs is very weak in ATF4 peaks, suggesting that in the case of the C/EBP dimer motif, C/EBP transcription factors known to interact with TRIB3 [32,34] may mediate TRIB3 association to chromatin, while in the case of the AP1 motif, the protein which might mediate TRIB3 binding to chromatin remains unknown. In addition to ATF4 and the other bZIP family members mentioned above, TRIB3 has been reported to interact and modulate the activity of several transcription factors, including NF-κB [33,53], a factor implicated in bortezomib-evoked multiple myeloma cell death [54] and PPARγ [35]. However, in our study, we detected no enrichment for NF-κB or PPARγ motifs in the ChIP-Seq data for TRIB3. This may be dependent on the cell type and experimental conditions being studied.

The results of ChIP-Seq analysis reveal that TRIB3 resides in the promoter–TSS of several hundred ATF4-binding genes that belong to functional categories of ER stress response, amino acid biosynthetic processes and cell death signaling, and TRIB3 binding to the chromatin loci is greatly elevated in response bortezomib exposure. We [51,52] and others [15] have shown that the downregulation of TRIB3 increases the ATF4-mediated gene expression in a variety of stresses. Genes may be regulated simultaneously by several transcription factors, conveying signals from different pathways, which may mask or compensate the changes in each other’s activities. For example, during ER stress, *DDIT3* expression is augmented by two UPR branches (ATF4 and ATF6) [55], and the expression of *SESN2* may be controlled by ATF4, XBP1 and p53 [56]. As revealed by our experiments with hepatoma cells exposed to bortezomib along with nelfinavir or salubrinal, an overactivation of the eIF2α–ATF4 pathway perturbs other UPR branches and accelerates cell death. Thus, TRIB3, acting as a negative feedback inhibitor of ATF4, appears to optimize ATF4 transcriptional activity for cell survival in conditions of proteasome inhibition. In line with this, in bortezomib-treated cells, TRIB3 genetic disruption results in an increased activation of ATF6 and XBP1 branches of the UPR and promotes cell death. Concordantly, the enforced expression of TRIB3 reduces bortezomib-evoked cell death, suggesting that constraining ATF4 activity delays initiation of the pro-death phase of UPR.

The cellular response to ER overload has been viewed as dual-faceted: a mild stress induces the transcription of genes which promote cellular adaptation to the altered conditions and support cell survival, while a strong, long-lasting stress leads to the activation of genes which promote cell death [23]. The switch from adaptive to pro-death ER stress response is thought to be linked to the high expression level and activity of DDIT3 [8,23]. In HepG2 cells, bortezomib exposure significantly upregulates *DDIT3* mRNA after 10 h and the expression is further increased at 20 h. Co-treatment of cells with bortezomib and nelfinavir upregulates *DDIT3* even more, which is consistent with DDIT3 serving a pro-death role. In line with this, we found that TRIB3, acting as a pro-survival protein during bortezomib stress and a negative regulator of C/EBP–ATF motif-mediated transcription, binds to the *DDIT3* promoter in bortezomib-exposed cells. In agreement with our findings, bortezomib-evoked apoptosis correlates with the induction of *DDIT3* expression in osteosarcoma cells [57] and *DDIT3* silencing inhibits death of non-small cell lung cancer and multiple myeloma cells co-treated with nelfinavir and bortezomib [58].

In addition to *DDIT3*, the ChIP-Seq results revealed also other genes harboring ATF4 and TRIB3 binding sites in bortezomib-treated HepG2 cells and known molecular functions related to cell death, including *DDIT4*, *JMY* and *ATF3*. DDIT4, the promoter region of which displays one of the strongest signals of TRIB3 binding, serves as a suppressor of mTORC1, and sustained DDIT4 expression during stress was found to decrease cell survival [59,60,61,62]. JMY, a cofactor of p53, increases p53-dependent transcription and apoptosis, and directs p53 to its pro-apoptotic targets during stress [63]. ATF3, a transcription factor with a pro-death role in several stressful situations, has been reported to positively regulate apoptosis-inducing genes, such as DR5 and NOXA, and ATF3 knockdown increases the viability of multiple myeloma cells treated by bortezomib [64]. Hence, multiple ATF4 target genes are co-bound by TRIB3 and might participate in the regulation of cell death in response to bortezomib treatment in hepatocellular carcinoma cells. Previous studies have reported that proteasome expression and activity is upregulated in tumors [18,21], and increased expression of proteasome components [19,20] as well as changes in subunit composition that increase the abundance of proteasomes less sensitive to inhibition [22] may influence the sensitivity of cancer cells to bortezomib. Therefore, we also analyzed the mRNA expression of proteasome components. While we note that bortezomib markedly induces many proteasome complex genes, this induction does not appear to be affected by the addition of pharmacological compounds modulating the eIF2α–ATF4 pathway. Similarly, in the absence of bortezomib, we observed no effect of ISRIB or nelfinavir on proteasome complex gene expression. However, it is still possible that the upregulation of proteasome component(s) via a currently undescribed post-transcriptional mechanism participates in the elevation of bortezomib resistance evoked by the eIF2α–ATF4 pathway inhibition.

## 4. Materials and Methods

### 4.1. Cell Culture and Treatment

HepG2 cells (ATCC) were maintained in Dulbecco’s Modified Eagle Medium (DMEM; 4.5 g/L D-glucose) supplemented with 10% fetal bovine serum and 1× penicillin-streptomycin at 37 °C in a 5% CO_2_ atmosphere (all medium components from Gibco, Paisley, UK). To perform chemical compound treatments, the growth medium was aspirated from the cells and replaced with fresh complete growth medium (described above) supplemented with the indicated concentration of chemical compound (or with vehicle as control). Bortezomib, nelfinavir (mesylate) and salubrinal were purchased from Cayman Chemical, and ISRIB and sodium arsenite from Sigma (St. Louis, MO, USA).

### 4.2. Plasmid Construction

To tag endogenous TRIB3 protein with a 3xFlag epitope, the homologous recombination donor plasmid TRIB3-pFETCh was constructed by inserting 0.94 kb 5′- and 0.96 kb 3′-homology arms flanking the human *TRIB3* gene stop codon into the plasmid pFETCh-Donor [36] (Addgene plasmid #63934). The homology arm DNA fragments were generated by PCR amplification of human genomic DNA using the primer pairs #942/#943 and #944/#945 (oligonucleotide sequences are shown in Table S4). To direct Cas9 nuclease activity near the *TRIB3* stop codon, CRISPR gRNA sequences were designed and inserted into the Cas9 expression plasmid pSpCas9(BB)-2A-GFP (PX458) [65] (Addgene plasmid #48138). Two alternative gRNA variants were prepared using the oligonucleotide pairs #946/#947 and #948/#949 (Table S4) and individually cloned into the vector, to obtain plasmids hTRIB3-HDR-guide1 and hTRIB3-HDR-guide2, respectively.

To generate the construct for plasmid-based expression of Flag-tagged TRIB3 (TRIB3-Flag-pQM), the coding region of human *TRIB3* cDNA extending from start codon to pre-stop codon was fused in-frame at the C-terminus to the pFETCh vector segment encoding 3xFlag, P2A and neomycin resistance gene, and inserted into the mammalian expression vector pQM-CTag/A (Quattromed, Tartu, Estonia) under the control of the CMV promoter. To prepare a corresponding plasmid expressing untagged (wild type) TRIB3 protein (TRIB3-pQM), the coding region of human *TRIB3* cDNA was inserted into the pQM-CTag/A vector.

To carry out CRISPR/Cas9-mediated disruption of the *TRIB3* gene, gRNA sequences targeting the 5′-end of the *TRIB3* coding region were inserted into the Cas9 expression plasmid pSpCas9(BB)-2A-GFP (PX458) [65] (Addgene plasmid #48138). Two gRNA variants were prepared using the oligonucleotide pairs #978/#979 and #980/#981 (Supplementary Table S4) and individually cloned into the vector, resulting in the plasmids hTRIB3-KO-guide1 and hTRIB3-KO-guide2, respectively. As a control region, a gRNA targeting the *AAVS1* locus was cloned into the PX458 vector using oligonucleotides #1023/#1024 (Table S4).

All constructed plasmids were verified by Sanger sequencing.

### 4.3. Epitope Tagging of Endogenous TRIB3 in Cell Culture

To generate the pool of HepG2 cells containing Flag-tagged endogenous TRIB3 (HepG2-TRIB3-Flag), cells were co-transfected with plasmids TRIB3-pFETCh and hTRIB3-HDR-guide1 by electroporation and stable transfectants were selected in the culture medium supplemented with 400 µg/mL G418 (Gibco) for 10 days, expanded as a pool, selected in the same conditions for additional 10 days, and used as a pool in experiment. To generate the clonal cell line HepG2-TRIB3-Flag clone#1, HepG2 cells were co-transfected with plasmids TRIB3-pFETCh, hTRIB3-HDR-guide1 and hTRIB3-HDR-guide2 using polyethylenimine (PEI-MAX 40,000; Polysciences Inc., Warrington, PA, USA, #24765), stable transfectants were selected using culture medium supplemented with 400 µg/mL G418, and individual clones were picked and expanded. In the individual clones as well as in the pool, homologous recombination (tagging of endogenous TRIB3) was verified by PCR and by Sanger sequencing, and the expression of TRIB3-Flag fusion protein was characterized using Western blotting and protein immunoprecipitation. HepG2-TRIB3-Flag pool and clone#1 were used for ChIP-Seq.

### 4.4. Chromatin Immunoprecipitation, ChIP-qPCR and Library Construction for ChIP-Seq

HepG2-TRIB3-Flag cells or unedited HepG2 cells were treated with bortezomib or vehicle (DMSO) and chromatin immunoprecipitation was performed as described previously [52], with some modifications. Briefly, cells were dual-crosslinked with 1.5 mM ethylene glycol bis(succinimidyl succinate) (Thermo Scientific Pierce #21565, Waltham, MA, USA) for 20 min, after which formaldehyde was added to a final concentration of 1.5% and cells were incubated for another 15 min at room temperature. The crosslinking reaction was stopped by adding 0.125 M glycine. Fixed cells were treated with nuclei isolation buffer (10 mM Hepes, pH 7.9, 85 mM KCl, 0.5% NP-40, Roche Complete protease inhibitor cocktail), resuspended in ChIP lysis buffer (50 mM Tris-HCl, pH 8.0, 0.3% SDS, 10 mM EDTA, protease inhibitors) and sonicated with Bioruptor Plus (Diagenode) for 20 cycles (30 s on, 30 s off) at 4 °C with power set to high. Sonicated chromatin was diluted 3-fold in ChIP dilution buffer (5 mM Tris-HCl, pH 8.0, 250 mM NaCl, 1.1% Triton X-100, protease inhibitors) and immunoprecipitated using mouse anti-Flag M2 monoclonal antibody (Sigma #F3165, St. Louis, MO, USA)**,** rabbit anti-ATF4 polyclonal antibody (Santa Cruz Biotechnology sc-200, Santa Cruz, CA, USA) or a negative control antibody, either mouse anti-E2Tag monoclonal antibody (Quattromed, Tartu, Estonia) or rabbit anti-GFP polyclonal antibody (Clontech #8367-1, Mountain View, CA, USA), with rotation overnight at 4 °C. Immunocomplexes were isolated by Protein G sepharose beads (Amersham Biosciences, Uppsala, Sweden) pre-blocked in 0.05% BSA. Subsequently, chromatin crosslinking was reversed and DNA was purified using QIAquick PCR Purification Kit (Qiagen, Hilden, Germany). Enrichment relative to input chromatin was quantified at selected genomics regions by real-time PCR using the primers presented in Table S4.

ChIP-seq libraries were prepared from 10 ng immunoprecipitated or input DNA using the NuGEN Ovation Ultralow V2 DNA-Seq library preparation kit (NuGEN Technologies, Redwood City, CA, USA). Library DNA was size selected with SPRI beads (Beckman Coulter, Brea, CA, USA) to retain 200–500 bp fragments. The libraries were sequenced on an Illumina NextSeq 500 using 76 bp single-end reads. The average sequencing depth was 27.1 million reads per library (range 23.7–31.5 million). The raw data from ChIP-Seq experiments has been deposited in NCBI GEO under the accession number GSE166958.

### 4.5. ChIP-Seq Data Analysis

Reads from ATF4 and TRIB3 ChIP-Seq experiments were processed with the nf-core ChIP-Seq pipeline (version 1.2.1; [66]) using the hg19 genome and BWA for alignment and MACS2 with the narrow peak profile for calling peaks and peak summits. Input DNA libraries were used as the background control. The aligned reads were used to create HOMER tag directories (version 4.11; [67]). Subsequently, HOMER mergePeaks was used to create a uniform peak set spanning the different IP and treatment conditions, the tag counts in these peaks were counted from each sample, and the ChIP-Seq signals were normalized to 10 million tags. HOMER annotatePeaks was used to analyze the gene context of peak regions. De novo motif finding was carried out using HOMER findMotifs with the full peak regions as the query (*size -given*) and automatic background region selection. Co-occurrence histograms (ChIP-Seq–ChIP-Seq or ChIP-Seq–motif) were calculated using HOMER annotatePeaks, centering the query regions on the peak summit coordinate. The position weight matrices of the query motifs were obtained from the HOMER motif library. The bin size for the histograms was 10 bp.

### 4.6. CRISPR/Cas9-Mediated Gene Disruption in Cell Culture

Cells were transfected with plasmid expressing Cas9 and gRNA (either hTRIB3-KO-guide1 and hTRIB3-KO-guide2 mixture, or AAVS1-KO-guide1) using the Lipofectamine 3000 transfection reagent (Thermo Scientific) and Opti-MEM I Reduced-Serum Medium (Thermo Scientific) supplemented with 3% FCS. 8 h post-transfection the medium was changed to complete growth medium. The next day, cells were seeded into either 96-well or 12-well plates and cultured for 24 h, after which the cells were used for luciferase reporter assays, for Trypan blue cell viability assays and for measuring mRNA or protein expression.

Genomic DNA was extracted from cells 24 h after transfection and the genomic region containing the CRISPR target site in the *TRIB3* gene was amplified with HOT FIREPol DNA Polymerase (Solis BioDyne, Tartu, Estonia) using primers #1050 and #1051 (Table S4). The PCR products were resolved on 1.8% agarose gel, stained with ethidium bromide and visualized by UV light to evaluate the fraction of alleles with a targeted deletion (excision of the gRNA1–gRNA2 fragment).

### 4.7. RNA-Seq Library Preparation and Sequencing

Total RNA from four biological replicates per condition was extracted using the RNeasy Mini Kit (Qiagen) according to manufacturer’s protocol for cultured cells, including on-column DNase I digestion. RNA yield and purity were measured on a NanoDrop 1000 Spectrophotometer (Thermo Scientific) to verify an A260/A280 nm ratio >1.8 and A260/A230 nm ratio ~2. Libraries were prepared from 250 ng of total RNA per sample using the Lexogen QuantSeq 3′ mRNA-Seq Library Prep Kit for Illumina (FWD) according to the manufacturer’s instructions.

Libraries were quantified with the Qubit dsDNA HS assay kit (Life Technologies, Waltham, MA, USA) and combined in equimolar ratio. The pool of libraries was sequenced at the Estonian Genome Center Core Facility using the Illumina NextSeq500 platform with 80 bp single-end reads. The average sequencing depth was 10.2 million reads per library (range 8.8–12.1 million). The raw data from RNA-Seq experiments has been deposited in NCBI GEO under the accession number GSE166923.

### 4.8. RNA-Seq Data Analysis

RNA-Seq reads were trimmed to remove Illumina adapter, poly(A) and low-quality bases using cutadapt (version 2.8; [68]). Subsequently, the reads were processed using the nf-core RNA-Seq pipeline (version 1.4.2; [66]) with trimming disabled and with STAR selected to align the reads to the hg19 human genome. Read counts in transcripts were determined according to Ensembl GRCh37/hg19 release 87 gene annotations for protein coding, lincRNA and antisense gene biotypes. Lowly expressed genes were removed using the filterByExpr function of the EdgeR package (version 3.24.3; [69]) requiring minimum count 5 and minimum total count 25. DESeq2 (version 1.22.2; [70]) was used to study differential gene expression between conditions. To quantify differential expression at the level of predefined gene sets, the target genes of individual UPR pathway branches were obtained from [24] and the branch-level activities were calculated and statistically evaluated using QuSAGE (version 2.16.1; [25]). For gene ontology over-representation analysis of gene lists, the g:Profiler tool (database release 22 July 2020; [71]) was used with GO Biological Process as the data source. To find enriched transcription factor binding motifs for lists of differentially expressed genes, i-cisTarget [72] was used (database version 6.0; search region TSS ± 10 kb).

### 4.9. Processing of Publicly Available Data Sets

ChIP-Seq data for H3K27ac and p300 in HepG2 cells was obtained from the ENCODE project [41] as BAM files aligned to hg19 genome (ENCODE file accessions ENCFF638JUP and ENCFF208LPX for H3K27ac, and ENCFF373JKP and ENCFF581OIB for p300). Replicates were combined into one HOMER tag directory. RNA-Seq based *TRIB3* gene expression levels for human cancer cell lines were obtained from the DepMap portal (release 20Q3; [29]) as transcripts per million and transformed to log_2_(1 + TPM). *TRIB3* RNA-Seq data for human tumors of different tissue origin and tumor type was obtained from The Cancer Genome Atlas (TCGA) portal [31] and filtered to retain only primary tumor samples. Patient survival data for hepatocellular carcinoma were obtained from TCGA and the samples were classified by *TRIB3* expression level into high (top 33%) and low (bottom 33%) expression of *TRIB3* and analyzed for differences in survival time using OncoLnc [73]. Drug sensitivity data for human cancer cell lines was obtained from two screening projects, CTRPv2 [27] and GDSC2 [28], as areas under concentration-response curves (AUCs) and filtered to retain only hepatic cancer cell lines.

### 4.10. Cell Viability Assays

HepG2 cell viability after drug exposure was determined using Alamar Blue (resazurin; Acros Organics) metabolic assay. 2 × 10^4^ cells/well were seeded in triplicate into 96-well plates and incubated for 24 h prior to the indicated treatments, resulting in a cell confluency of approximately 70% at the start of compound treatment. For cell viability assessment, the treatment medium was replaced with DMEM containing 0.03 mg/mL resazurin and the cells were incubated further for 3 h at 37 °C and 5% CO_2_. Fluorescence was measured on a GENios Plus (Tecan, Männedorf, Switzerland) microplate reader (excitation, 540 nm; emission, 595 nm). The background fluorescence of resazurin solution without cells was subtracted from all samples and the percentage of cell viability was calculated relative to results obtained for cells treated with vehicle (DMSO).

To evaluate the effect of TRIB3 on cell viability, the Trypan blue dye exclusion method was used [74]. HepG2 cells were transfected with either Cas9–gRNA plasmids (TRIB3-g1-PX458 and TRIB3-g2-PX458 mixture, or AAVS1-PX458) or TRIB3 overexpression plasmid (TRIB3-pCG; [12]) and empty control vector (pCG) using the Lipofectamine 3000 transfection reagent (Thermo Scientific) as described above. The next day, cells were trypsinized and seeded at a density of 2 × 10^4^ cells/well into 96-well plates, cultured for 24 h and treated as indicated. Cells were then collected, stained with 0.1% Trypan blue solution in PBS and viable and non-viable (dye-accumulating) cells were counted in a hemocytometer.

### 4.11. RT-PCR and RT-qPCR

Total RNA was extracted from cells using the TRIzol reagent (Invitrogen, Waltham, MA, USA), quantified with the NanoDrop 1000 spectrophotometer (Thermo Scientific), treated with DNase I and subjected to cDNA synthesis using FIREScript reverse transcriptase (Solis BioDyne, Tartu, Estonia) according to the manufacturer’s instructions. Real-time quantitative PCR was performed as described previously [75]. The mRNA encoding glyceraldehyde-3-phosphate dehydrogenase (GAPDH) was used as the endogenous reference for gene expression normalization. The sequences of RT-qPCR primers are shown in Supplementary Table S4.

*XBP1* splicing was detected by RT-PCR using HOT FIREPol DNA Polymerase (Solis BioDyne) and primers that span the IRE1-mediated splicing event on the *XBP1* mRNA (Table S4). For control reactions, primers targeting *GAPDH* mRNA were used (Table S4). The PCR products were resolved on 3.5% agarose gel, stained with ethidium bromide and visualized by UV light.

### 4.12. Dual-Luciferase Reporter Assay

Transcriptional activity reporter assays were performed using a protocol adapted from [52]. Cells grown in 96-well plates were co-transfected in duplicate wells with 70 ng of firefly luciferase plasmid (either pGL3-Basic or reporter construct with 3 × 33 bp repeats containing C/EBP-ATF response element (plasmid -7131/-7033 from [12])) and 10 ng of *Renilla* luciferase plasmid (pRL-TK; Promega) using polyethylenimine (PEI-MAX 40,000; Polysciences Inc. #24765). To compare TRIB3 and TRIB3-Flag protein abilities to inhibit transcription from C/EBP-ATF sites, the expression plasmids for human TRIB3 with or without Flag tag in C-terminus (TRIB3-Flag-pQM or TRIB3-pQM, respectively) and empty vector pQM were added to the transfection mixture. Transfected cells were cultured for 20 h, after which the cells were treated with the indicated compounds for 10 h. Firefly and *Renilla* luciferase activities were measured using the dual-luciferase assay (Promega, Madison, WI, USA) as described previously [12,74]. The firefly luciferase activity was normalized to the *Renilla* luciferase activity for each sample, and the activities of reporter construct with 3 × 33 bp repeats are presented relative to the activity of the promoterless pGL3-Basic plasmid in the same experimental conditions.

### 4.13. Western Blotting

Immunoblotting was carried out as described previously [76]. The following antibodies were used: mouse anti-Flag M2 monoclonal antibody (1:1500 dilution; Sigma #F3165), rabbit anti-ATF4 polyclonal antibody (1:5000 dilution; Santa Cruz Biotechnology sc-200, Santa Cruz, CA, USA) and rabbit anti-β-Tubulin polyclonal antibody (1:2500 dilution; Abcam #ab6046, Cambridge, UK). Depending on the species of the primary antibody, the secondary antibody used was either horseradish peroxidase-conjugated goat anti-mouse IgG secondary antibody (1:10,000 dilution; Thermo Scientific #31430) or horseradish peroxidase-conjugated goat anti-rabbit IgG secondary antibody (1:5000 dilution; Cell Signaling Technology #7074, Danvers, MA, USA). Blots were treated with Immobilon chemiluminescent reagent (EMD Millipore, Burlington, MA, USA) and proteins were visualized either on autoradiography film or with ChemiDoc XRS+ detection system (BioRad). Uncropped western blot images are presented in Figures S2 and S3.

### 4.14. Protein Immunoprecipitation

Immunoprecipitation of protein complexes was performed as described previously [30], with minor modifications. Cells were lysed in buffer L (50 mM Tris-HCl, pH 7.5, 150 mM NaCl, 1% Triton X-100, 0.1% sodium deoxycholate, 0.1% SDS, 1 mM EDTA, 10 mM NaF, Roche Complete protease inhibitor cocktail), incubated on ice for 30 min and sonicated with a Bioruptor Plus (Diagenode, Denville, NJ, USA) for 10 cycles (30 s on, 30 s off) at 4 °C with power set to high. Lysates were pre-cleared by centrifugation at 15,000 rpm for 15 min and immunoprecipitated using either mouse anti-Flag M2 monoclonal antibody (1:400 dilution; Sigma #F3165), goat anti-Flag (OctA-Probe D-8) polyclonal antibody (1:30 dilution; Santa Cruz Biotechnology sc-807-G), rabbit anti-TRIB3 polyclonal antibody (1:300 dilution; Calbiochem #ST1032, Burlington, MA, USA) or, as a negative control antibody, mouse anti-E2Tag monoclonal antibody (1:120 dilution; Quattromed) with rotation overnight at 4 °C. The immunocomplexes were collected from the lysate with Protein G sepharose beads (Amersham Biosciences) and eluted in SDS gel sample buffer. The eluted samples were resolved on 10% SDS-PAGE and analyzed by Western blotting as described above.

## 5. Conclusions

By using combinations of chemical treatments, we find that eIF2α–ATF4 pathway activity level is a factor affecting hepatoma cell sensitivity to proteasome inhibition. The ability of the protein TRIB3 to bind a large portion of genomic sites occupied by ATF4 and restrict ATF4-dependent transcription promotes hepatoma cells resistance to bortezomib treatment.

## Data Availability

The raw data from high-throughput sequencing experiments is publicly available through NCBI GEO (www.ncbi.nlm.nih.gov/geo) under the accession numbers GSE166958 (ChIP-Seq) and GSE166923 (RNA-Seq).

## Supplementary Materials

The following are available online at https://www.mdpi.com/article/10.3390/cancers13102341/s1, Figure S1: Expression of proteasome complex (GO:0000502) genes in HepG2 cells treated with bortezomib (Btz), nelfinavir (Nelf) and ISRIB, Figure S2: Uncropped Western blot images for Figure 1D (A), Figure 5C (B), Figure 5D left panels (C) and Figure 5D right panels (D), Figure S3: Uncropped Western blot images for Figure 5E (A), Figure 5F left panels (B), Figure 5F right panels (C), Figure 8C (D) and Figure 8D (E), Table S1: RNA-Seq differential gene expression analysis results, Table S2: (A) ChIP-Seq peaks for TRIB3 and ATF4 in HepG2 cells, (B) Genes with TRIB3 ChIP-Seq peaks within <2 kb of a transcription start site (TSS), Table S3: Motif enrichment results for ChIP-Seq peaks, Table S4: Oligonucleotides used for plasmid construction and PCR, RT-PCR, RT-qPCR and ChIP-qPCR experiments.

## Abbreviations

ISR: integrated stress response; TSS, transcription start site; UPR, unfolded protein response.
