# Supplementary material for: Pharmacological or TRIB3-Mediated Suppression of ATF4 Transcriptional Activity Promotes Hepatoma Cell Resistance to Proteasome Inhibitor Bortezomib
Source: Cancers (Basel). 2021 May 12;13(10):2341. doi: 10.3390/cancers13102341 (PMC8150958; doi:10.3390/cancers13102341)
Supplement: Supplementary file 1 [file cancers-13-02341-s001.zip › Supplementary Table S3.pdf]

Supplementary Table S3. Motif enrichment results for ChIP-Seq peaks

(A) HOMER *de novo* Motif Results – TRIB3 peaks (bortezomib treated)

Total target sequences = 7635
Total background sequences = 42011

Rank	Motif	P-value	% of Targets	% of Background	Best Match/Details (Match Score)
1	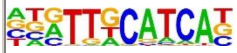	1e-3283	53.01%	4.37%	Chop(bZIP)/MEF-Chop-ChIP-Seq(GSE35681)/Homer(0.98)<br>Atf4(bZIP)/MEF-Atf4-ChIP-Seq(GSE35681)/Homer(0.98)<br>ATF4/MA0833.1/Jaspar(0.92)
2	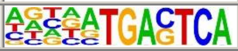	1e-508	29.23%	9.57%	BATF(bZIP)/Th17-BATF-ChIP-Seq(GSE39756)/Homer(0.976)
3	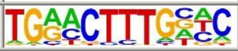	1e-162	14.98%	6.21%	HNF4G/MA0484.1/Jaspar(0.959)
4	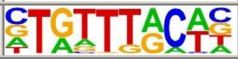	1e-154	42.38%	28.17%	FOXP1/MA0481.2/Jaspar(0.907)
5	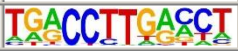	1e-99	14.26%	7.22%	Nr5a2/MA0505.1/Jaspar(0.949)
6	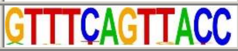	1e-96	1.35%	0.06%	ISRE(IRF)/ThioMac-LPS-Expression(GSE23622)/Homer(0.753)
7	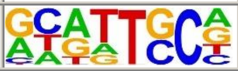	1e-93	20.41%	12.10%	TEAD1(TEAD)/HepG2-TEAD1-ChIP-Seq(Encode)/Homer(0.793)
8	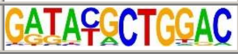	1e-77	1.32%	0.09%	ZNF416(Zf)/HEK293-ZNF416.GFP-ChIP-Seq(GSE58341)/Homer(0.591)
9	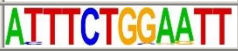	1e-76	1.36%	0.10%	TEAD3(TEA)/HepG2-TEAD3-ChIP-Seq(Encode)/Homer(0.688)
10	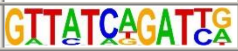	1e-74	0.71%	0.01%	Pax7(Paired,Homeobox),long/Myoblast-Pax7-ChIP-Seq(GSE25064)/Homer(0.692)
11	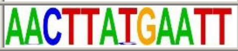	1e-64	0.67%	0.02%	PB0068.1_Sox1_1/Jaspar(0.649)
12	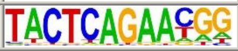	1e-61	1.56%	0.21%	STAT5(Stat)/mCD4+-Stat5-ChIP-Seq(GSE12346)/Homer(0.714)
13	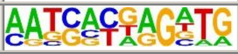	1e-56	0.58%	0.01%	PB0098.1_Zfp410_1/Jaspar(0.586)
14	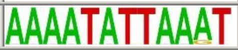	1e-54	1.24%	0.14%	Arid3a/MA0151.1/Jaspar(0.673)
15	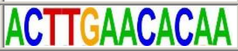	1e-53	0.45%	0.01%	Oct4:Sox17(POU,Homeobox,HMG)/F9-Sox17-ChIP-Seq(GSE44553)/Homer(0.661)

(B) HOMER *de novo* Motif Results – TRIB3 peaks (untreated)

Total target sequences = 487
Total background sequences = 48823
* - possible false positive (HOMER warning)

Rank	Motif	P-value	% of Targets	% of Background	Best Match/Details (Match Score)
1	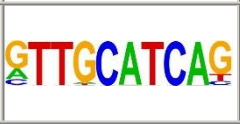	1e-329	61.19%	2.71%	Atf4(bZIP)/MEF-Atf4-ChIP-Seq(GSE35681)/Homer(0.95)<br>Chop(bZIP)/MEF-Chop-ChIP-Seq(GSE35681)/Homer(0.95)<br>ATF4/MA0833.1/Jaspar(0.88)
2	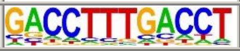	1e-21	39.01%	19.91%	Nr2f6/MA0677.1/Jaspar(0.924)
3	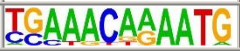	1e-17	6.98%	0.96%	PB0123.1_Foxl1_2/Jaspar(0.620)
4	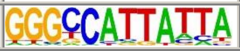	1e-17	6.78%	0.94%	Nanog(Homeobox)/mES-Nanog-ChIP-Seq(GSE11724)/Homer(0.686)
5	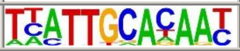	1e-16	5.54%	0.61%	CEBPA/MA0102.3/Jaspar(0.913)
6	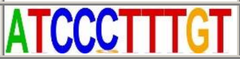	1e-15	3.90%	0.27%	Sox2/MA0143.3/Jaspar(0.788)
7	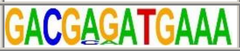	1e-15	1.64%	0.01%	Tcf7(HMG)/GM12878-TCF7-ChIP-Seq(Encode)/Homer(0.616)
8	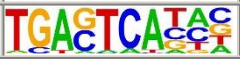	1e-13	19.71%	8.57%	BATF(bZIP)/Th17-BATF-ChIP-Seq(GSE39756)/Homer(0.980)
9	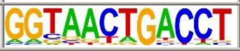	1e-12	6.78%	1.37%	FXR(NR),IR1/Liver-FXR-ChIP-Seq(Chong_et_al.)/Homer(0.794)
10	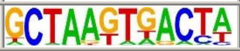	1e-12	3.90%	0.41%	Isl1(Homeobox)/Neuron-Isl1-ChIP-Seq(GSE31456)/Homer(0.688)
11 *	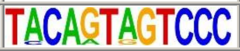	1e-11	2.05%	0.07%	PB0051.1_Osr2_1/Jaspar(0.648)
12 *	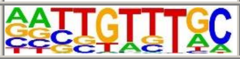	1e-11	13.76%	5.36%	Foxo3(Forkhead)/U2OS-Foxo3-ChIP-Seq(E-MTAB-2701)/Homer(0.821)
13 *	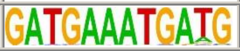	1e-11	1.64%	0.03%	Chop(bZIP)/MEF-Chop-ChIP-Seq(GSE35681)/Homer(0.674)
14 *	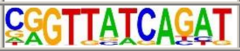	1e-11	1.64%	0.03%	PB0109.1_Bbx_2/Jaspar(0.705)
15 *	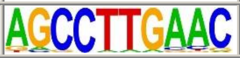	1e-10	12.53%	4.92%	RAR:RXR(NR),DR5/ES-RAR-ChIP-Seq(GSE56893)/Homer(0.754)

(C) HOMER *de novo* Motif Results – ATF4 peaks (bortezomib treated)

Total target sequences = 6856
Total background sequences = 42868

Rank	Motif	P-value	% of Targets	% of Background	Best Match/Details (Match Score)
1	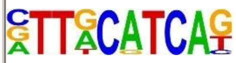	1e-5226	76.37%	4.99%	Chop(bZIP)/MEF-Chop-ChIP-Seq(GSE35681)/Homer(0.98)<br>Atf4(bZIP)/MEF-Atf4-ChIP-Seq(GSE35681)/Homer(0.97)<br>ATF4/MA0833.1/Jaspar(0.89)
2	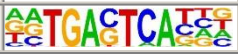	1e-270	18.61%	6.15%	Atf3(bZIP)/GBM-ATF3-ChIP-Seq(GSE33912)/Homer(0.994)
3	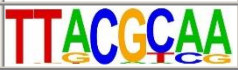	1e-267	42.05%	22.98%	CEBPB/MA0466.2/Jaspar(0.924)
4	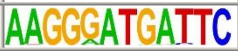	1e-153	1.76%	0.04%	JUN(var.2)/MA0489.1/Jaspar(0.668)
5	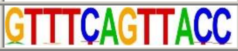	1e-98	1.55%	0.07%	ISRE(IRF)/ThioMac-LPS-Expression(GSE23622)/Homer(0.759)
6	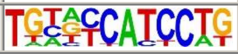	1e-88	5.63%	1.69%	FOSL2/MA0478.1/Jaspar(0.672)
7	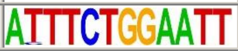	1e-83	1.24%	0.05%	TEAD3(TEA)/HepG2-TEAD3-ChIP-Seq(Encode)/Homer(0.700)
8	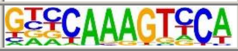	1e-81	12.62%	6.29%	HNF4G/MA0484.1/Jaspar(0.943)
9	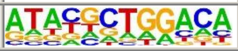	1e-77	1.47%	0.10%	ZNF416(Zf)/HEK293-ZNF416.GFP-ChIP-Seq(GSE58341)/Homer(0.627)
10	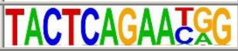	1e-64	1.41%	0.13%	STAT5(Stat)/mCD4+-Stat5-ChIP-Seq(GSE12346)/Homer(0.659)
11	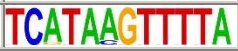	1e-62	0.80%	0.02%	BHLHE23/MA0817.1/Jaspar(0.620)
12	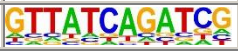	1e-60	0.89%	0.04%	PB0022.1_Gata5_1/Jaspar(0.701)
13	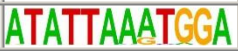	1e-58	1.21%	0.10%	Arid3a/MA0151.1/Jaspar(0.672)
14	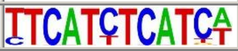	1e-54	0.76%	0.03%	PH0014.1_Cphx/Jaspar(0.653)
15	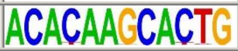	1e-51	0.73%	0.03%	PB0195.1_Zbtb3_2/Jaspar(0.621)

(D) HOMER Known Motif Results – TRIB3 peaks (bortezomib treated)

Total Target Sequences = 7635
Total Background Sequences = 42012

Rank	Motif	Name	P-value	% of Targets Sequences with Motif	% of Background Sequences with Motif
1	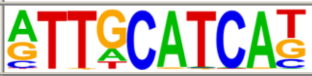	Atf4(bZIP)/MEF-Atf4-ChIP-Seq(GSE35681)/Homer	1e-3183	65.72%	9.19%
2	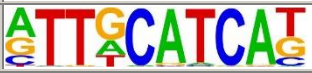	Chop(bZIP)/MEF-Chop-ChIP-Seq(GSE35681)/Homer	1e-3132	60.92%	7.44%
3	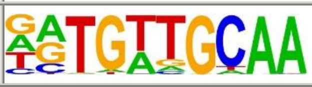	CEBP:AP1(bZIP)/ThioMac-CEBPb-ChIP-Seq(GSE21512)/Homer	1e-1940	73.82%	22.78%
4	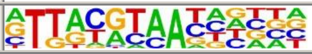	NFIL3(bZIP)/HepG2-NFIL3-ChIP-Seq(Encode)/Homer	1e-963	55.10%	20.43%
5	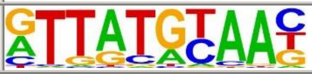	HLF(bZIP)/HSC-HLF.Flag-ChIP-Seq(GSE69817)/Homer	1e-904	61.93%	26.66%
6	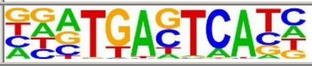	Fra1(bZIP)/BT549-Fra1-ChIP-Seq(GSE46166)/Homer	1e-550	36.88%	13.82%
7	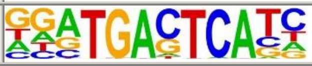	Fra2(bZIP)/Striatum-Fra2-ChIP-Seq(GSE43429)/Homer	1e-550	32.94%	11.28%
8	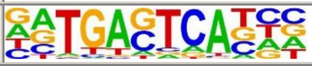	Fosl2(bZIP)/3T3L1-Fosl2-ChIP-Seq(GSE56872)/Homer	1e-537	25.63%	7.18%
9	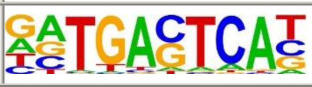	JunB(bZIP)/DendriticCells-Junb-ChIP-Seq(GSE36099)/Homer	1e-537	36.02%	13.45%
10	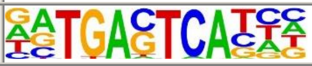	Atf3(bZIP)/GBM-ATF3-ChIP-Seq(GSE33912)/Homer	1e-511	39.96%	16.60%
11	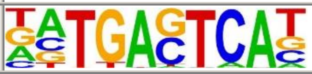	BATF(bZIP)/Th17-BATF-ChIP-Seq(GSE39756)/Homer	1e-493	39.19%	16.37%
12	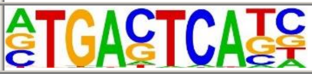	AP-1(bZIP)/ThioMac-PU.1-ChIP-Seq(GSE21512)/Homer	1e-477	41.64%	18.46%
13	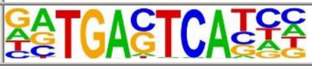	Jun-AP1(bZIP)/K562-cJun-ChIP-Seq(GSE31477)/Homer	1e-471	20.39%	5.14%
14	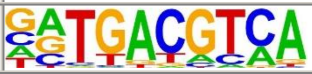	Atf1(bZIP)/K562-ATF1-ChIP-Seq(GSE31477)/Homer	1e-261	39.20%	21.68%
15	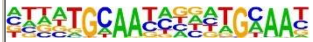	CEBP:CEBP(bZIP)/MEF-Chop-ChIP-Seq(GSE35681)/Homer	1e-259	16.49%	5.51%

(E) HOMER Known Motif Results – TRIB3 peaks (untreated)

Total Target Sequences = 487
Total Background Sequences = 48863

Rank	Motif	Name	P-value	% of Targets Sequences with Motif	% of Background Sequences with Motif
1	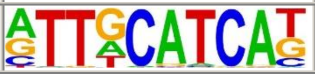	Chop(bZIP)/MEF-Chop-ChIP-Seq(GSE35681)/Homer	1e-318	82.14%	9.32%
2	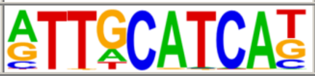	Atf4(bZIP)/MEF-Atf4-ChIP-Seq(GSE35681)/Homer	1e-301	84.80%	11.58%
3	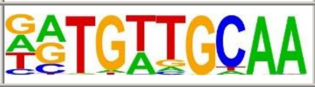	CEBP:AP1(bZIP)/ThioMac-CEBPb-ChIP-Seq(GSE21512)/Homer	1e-180	89.12%	27.27%
4	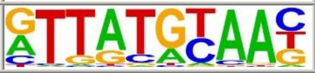	HLF(bZIP)/HSC-HLF.Flag-ChIP-Seq(GSE69817)/Homer	1e-81	73.31%	30.97%
5	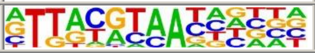	NFIL3(bZIP)/HepG2-NFIL3-ChIP-Seq(Encode)/Homer	1e-68	61.81%	24.30%
6	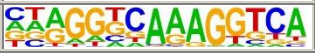	PPARa(NR),DR1/Liver-Ppara-ChIP-Seq(GSE47954)/Homer	1e-14	50.31%	32.82%
7	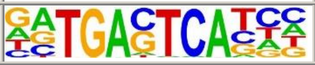	Jun-AP1(bZIP)/K562-cJun-ChIP-Seq(GSE31477)/Homer	1e-14	16.84%	6.59%
8	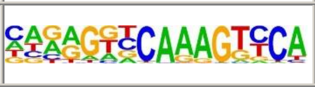	HNF4a(NR),DR1/HepG2-HNF4a-ChIP-Seq(GSE25021)/Homer	1e-13	27.72%	14.43%
9	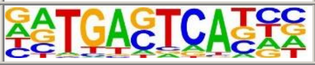	Fosl2(bZIP)/3T3L1-Fosl2-ChIP-Seq(GSE56872)/Homer	1e-13	20.33%	9.24%
10	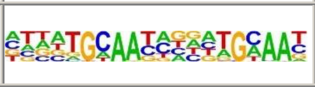	CEBP:CEBP(bZIP)/MEF-Chop-ChIP-Seq(GSE35681)/Homer	1e-12	16.22%	6.76%
11	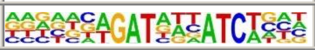	GATA(Zf),IR3/iTreg-Gata3-ChIP-Seq(GSE20898)/Homer	1e-12	16.63%	7.07%
12	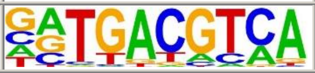	Atf1(bZIP)/K562-ATF1-ChIP-Seq(GSE31477)/Homer	1e-12	43.12%	28.09%
13	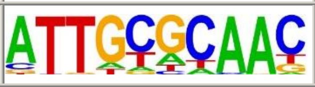	CEBP(bZIP)/ThioMac-CEBPb-ChIP-Seq(GSE21512)/Homer	1e-11	44.15%	29.04%
14	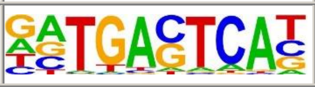	JunB(bZIP)/DendriticCells-Junb-ChIP-Seq(GSE36099)/Homer	1e-10	28.95%	16.76%
15	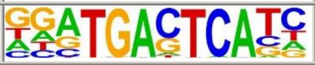	Fra2(bZIP)/Striatum-Fra2-ChIP-Seq(GSE43429)/Homer	1e-10	25.87%	14.41%

(F) HOMER Known Motif Results – ATF4 peaks (bortezomib treated)

Total Target Sequences = 6856
Total Background Sequences = 42881

Rank	Motif	Name	P-value	% of Targets Sequences with Motif	% of Background Sequences with Motif
1	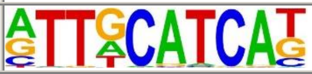	Chop(bZIP)/MEF-Chop-ChIP-Seq(GSE35681)/Homer	1e-4647	79.65%	7.63%
2	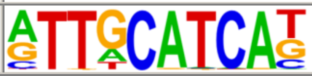	Atf4(bZIP)/MEF-Atf4-ChIP-Seq(GSE35681)/Homer	1e-4490	83.12%	9.64%
3	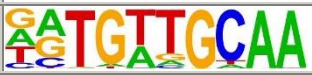	CEBP:AP1(bZIP)/ThioMac-CEBPb-ChIP-Seq(GSE21512)/Homer	1e-2676	86.30%	23.18%
4	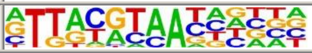	NFIL3(bZIP)/HepG2-NFIL3-ChIP-Seq(Encode)/Homer	1e-1304	65.18%	21.60%
5	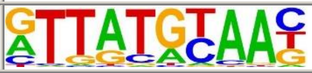	HLF(bZIP)/HSC-HLF.Flag-ChIP-Seq(GSE69817)/Homer	1e-1279	72.71%	28.00%
6	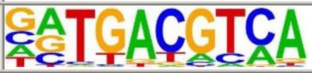	Atf1(bZIP)/K562-ATF1-ChIP-Seq(GSE31477)/Homer	1e-321	42.11%	21.44%
7	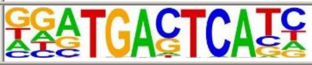	Fra2(bZIP)/Striatum-Fra2-ChIP-Seq(GSE43429)/Homer	1e-281	26.44%	10.86%
8	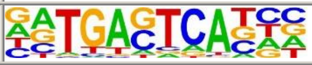	Fosl2(bZIP)/3T3L1-Fosl2-ChIP-Seq(GSE56872)/Homer	1e-270	19.59%	6.72%
9	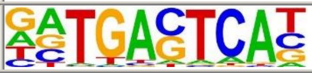	JunB(bZIP)/DendriticCells-Junb-ChIP-Seq(GSE36099)/Homer	1e-261	29.04%	13.11%
10	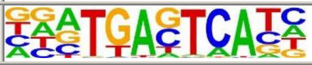	Fra1(bZIP)/BT549-Fra1-ChIP-Seq(GSE46166)/Homer	1e-259	29.67%	13.61%
11	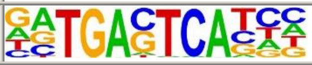	Jun-AP1(bZIP)/K562-cJun-ChIP-Seq(GSE31477)/Homer	1e-237	15.26%	4.74%
12	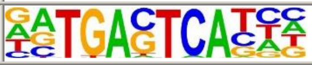	Atf3(bZIP)/GBM-ATF3-ChIP-Seq(GSE33912)/Homer	1e-236	32.48%	16.30%
13	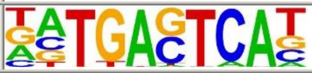	BATF(bZIP)/Th17-BATF-ChIP-Seq(GSE39756)/Homer	1e-234	31.94%	15.96%
14	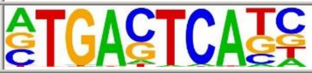	AP-1(bZIP)/ThioMac-PU.1-ChIP-Seq(GSE21512)/Homer	1e-233	34.32%	17.82%
15	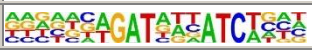	GATA(Zf),IR3/iTreg-Gata3-ChIP-Seq(GSE20898)/Homer	1e-223	17.81%	6.45%
