# Supplementary material for: Pharmacological or TRIB3-Mediated Suppression of ATF4 Transcriptional Activity Promotes Hepatoma Cell Resistance to Proteasome Inhibitor Bortezomib
Source: Cancers (Basel). 2021 May 12;13(10):2341. doi: 10.3390/cancers13102341 (PMC8150958; doi:10.3390/cancers13102341)
Supplement: Supplementary file 1 [file cancers-13-02341-s001.zip › Supplementary Table S4.pdf]

**Supplementary Table S4.** Oligonucleotides used for plasmid construction and PCR, RT-PCR, RT-qPCR and ChIP-qPCR experiments.

**Oligonucleotides used for preparing the pFETCh-TRIB3 construct**

PCR primers for the amplification of the 5′-homology arm:

Oligo hTRIB3-5FL-S (#942): 5′-CCTGGTCTCAAGCTGGGTCAAGGGCCAGGAACAC-3′

Oligo hTRIB3-5FL-AS (#943): 5′-CCTGGTCTCTGCTCCCGCCATACAGAACCACTTCTCTGTCTCCCTC-3′

PCR primers for the amplification of the 3′-homology arm:

Oligo hTRIB3-3FL-S (#944): 5′-CCTGAAGACATCATCACGCTCAGCTGCCAACAGTGG-3′

Oligo hTRIB3-3FL-AS (#945): 5′-CCTGAAGACATCAAGGCCTTTCACCATGGGGGCCAG-3′

**Oligonucleotides used for preparing the CRISPR gRNA constructs for 3xFlag cassette insertion into the endogenous *TRIB3* locus**

Oligonucleotides for hTRIB3-HDR-guide1:

Oligo hTRIB3-HDR-guide1A (#946): 5′-CACCGCAGCTGAGCGTGTAGTAGGG-3′

Oligo hTRIB3- HDR-guide1B (#947): 5′-AAACCCCTACTACACGCTCAGCTGC-3′

Oligonucleotides for hTRIB3-HDR-guide2:

Oligo hTRIB3-HDR-guide2A (#948): 5′-CACCGAGCTGAGCGTGTAGTAGGG-3′

Oligo hTRIB3-HDR-guide2B (#949): 5′-AAACCCCTACTACACGCTCAGCTC-3′

## PCR primers for validation of 3xFlag cassette insertion into the endogenous *TRIB3* locus

PCR primers for 5′-region validation:

Oligo pFETCh-SEQ-P2A (#952): 5′-CAGCAGGCTGAAGTTAGTAGC-3′

Oligo hTRB3-vali-Int3-S (#956): 5′-CCACTGGACACCCAGGAAGTAATGC-3′

PCR primers for 3′-region validation:

Oligo pFETCh-Neo-3PRI-S (#955): 5′-ACCGCTTCCTCGTGCTTTAC-3′

Oligo hTRB3-vali-3FL-AS (#957): 5′-CAATGACCCCTGGCTTCTTCCTC-3′

## Oligonucleotides used for preparing CRISPR gRNA constructs to disrupt the human *TRIB3* gene

hTRIB3-KO-guide1:

Oligo hTRIB3-KO-guide1A (#978): 5′-CACCGCAGAGGGGTGGCTCGCATC-3′

Oligo hTRIB3-KO-guide1B (#979): 5′-AAACGATGCGAGCCACCCCTCTGC-3′

hTRIB3-KO-guide2:

Oligo hTRIB3-KO-guide2A (#980): 5′-CACCGACATAGGGCCCAAGACGGG-3′

Oligo hTRIB3-KO-guide2B (#981): 5′-AAACCCCGTCTTGGGCCCTATGTC-3′

## PCR primers used for validation of CRISPR/Cas9-mediated disruption of the *TRIB3* gene

Forward (#1050): 5′-CACCCCCTATAAATGTTGTGCCACGT-3′

Reverse (#1051): 5′-GCAGGTATACTCAGTGCCTGTAGGGC-3′

## <u>Oligonucleotides used for making CRISPR guide RNA construct targeting the AAVS1 locus (used as a control region) of the human genome</u>

<u>AAVS1-KO-guide1:</u>

Oligo AAVS1-KO-guide1A (#1023): 5′-CACCGTTAATGTGGCTCTGGTTCT-3′

Oligo AAVS1-KO-guide1B (#1024): 5′-AAACAGAACCAGAGCCACATTAAC-3′

## <u>Primer sequences used for RT-PCR, RT-qPCR and ChIP-qPCR experiments</u>

| Method | Target | Forward primer (5′→ 3′) | Reverse primer (5′→ 3′) |
|---|---|---|---|
| RT-PCR | *XBP1* splicing | AGCCAAGGGGAATGAAGTGAG | CTGGGTCCAAGTTGTCCAGAA |
| | *GAPDH* mRNA | TGCACCACCAACTGCTTAGC | GGCATGGACTGTGGTCATGAG |
| RT-qPCR | *TRIB3* mRNA | TTTGTCTTCGCTGACCGTGA | TGCTTGTCCCACAGGGAATC |
| | *ASNS* mRNA | ATCACTGTCGGGATGTACCC | CTTCAACAGAGTGGCAGCAA |
| | *SLC7A11* mRNA | GCAAGCACACTCCTCTACCA | GCACCTTGAAAGGACGATGC |
| | *MTHFD2* mRNA | CCATGTTACCGGCTACTCCA | GTAACAGTGGCATCACCTCCG |
| | *PSAT1* mRNA | CCGTGGTGATTGTCCGTGAT | ATCCACTCCAGAACCAAGCC |
| | *DDIT4* mRNA | GGTTTGACCGCTCCACGAG | GGTAAGCCGTGTCTTCCTCC |
| | *SESN2* mRNA | CCACTCAGAGAAGGTCCACG | GAGTCAGGTCATGTAGCGGG |
| | *DDIT3* mRNA | CCTGCAAGAGGTCCTGTCTT | GTGACCTCTGCTGGTTCTGG |
| | *HSPA5* mRNA | GAACACAGTGGTGCCTACCAA | AGGTGACTTCAATCTGTGGGAC |
| | *HSP90B1* mRNA | GTTTCCCGCGAGACTCTTCA | ACGTGTTCGATTCGAGTGGT |
| | *GAPDH* mRNA | TGCACCACCAACTGCTTAGC | GGCATGGACTGTGGTCATGAG |

| | | | |
|---|---|---|---|
| ChIP-qPCR | *DDIT4* promoter | CTGCCAGGCCAGATTTCCT | GCCATCCCGTGTTTCATCA |
| | *ASNS* promoter | AGGGATGTGGACAGCTTGAC | GCAGGCATGATGAAACTTCC |
| | *TRIB3* promoter | GGTCCCAGGTGCAGCGGATGCAG | CGGGTGATGCAAACCGGAGCTAATCAG |
| | *PSAT1* promoter | AATCTCGCGCGCTCCTTG | CATTGGTCGCGCTTCTGAG |
| | Neg. control region near *TRIB3* | GAGTCCCCAGCTGTGCTAAC | GTCCGAGTGAAAAAGGCGTA |
