# Supplementary material for: Pharmacological or TRIB3-Mediated Suppression of ATF4 Transcriptional Activity Promotes Hepatoma Cell Resistance to Proteasome Inhibitor Bortezomib
Source: Cancers (Basel). 2021 May 12;13(10):2341. doi: 10.3390/cancers13102341 (PMC8150958; doi:10.3390/cancers13102341)
Supplement: Supplementary file 1 [file cancers-13-02341-s001.zip › Supplementary Text S1 - Supplementary Material Descriptions.pdf]

Supplementary Figure Legends

Supplementary Figure S1. Expression of proteasome complex (GO:0000502) genes in HepG2 cells treated with bortezomib (Btz), nelfinavir (Nelf) and ISRIB. **(A)** Gene expression level heatmap. Each column corresponds to an RNA-Seq library. **(B)** Gene set activity calculated for the proteasome complex genes shown in panel A. The combined expression change was calculated using QuSAGE. The p values shown have been corrected using the Bonferroni–Holm method.

Supplementary Figure S2. Uncropped Western blot images for Figures 1D (A), 5C (B), 5D left panels (C) and 5D right panels (D). The value below the protein band represents the relative intensity determined by densitometric analysis using Image Lab software (BioRad).

Supplementary Figure S3. Uncropped Western blot images for Figures 5E (A), 5F left panels (B), 5F right panels (C), 8C (D) and 8D (E). The value below the protein band represents the relative intensity determined by densitometric analysis using Image Lab software (BioRad).

Supplementary Table Descriptions

Supplementary Table S1. RNA-Seq differential gene expression analysis results. The tables are sorted by effect direction and p value (most significantly upregulated at the top; most significantly downregulated at the bottom).

Supplementary Table S2. (A) ChIP-Seq peaks for TRIB3 and ATF4 in HepG2 cells. For each region, the presence of a peak is indicated for the conditions studied (TRIB3 untreated, TRIB3 bortezomib and ATF4 bortezomib). Peak annotations by HOMER are shown. Average ChIP-Seq signal is shown normalized to 10 million tags. **(B)** Genes with TRIB3 ChIP-Seq peaks within <2 kb of a transcription start site (TSS).

Supplementary Table S3. Motif enrichment results for ChIP-Seq peaks. **(A–C)** The HOMER *de novo* motif results for **(A)** TRIB3 peaks from bortezomib treatment, **(B)** TRIB3 peaks from control, and **(C)** ATF4 peaks from bortezomib treatment. **(D–F)** The HOMER known motif results for **(D)** TRIB3 peaks from bortezomib treatment, **(E)** TRIB3 peaks from control, and **(F)** ATF4 peaks from bortezomib treatment. In each case, the top 15 motif results by p value are shown.

Supplementary Table S4. Oligonucleotides used for plasmid construction and PCR, RT-PCR, RT-qPCR and ChIP-qPCR experiments.
