# Supplementary figures and images for: Pharmacological or TRIB3-Mediated Suppression of ATF4 Transcriptional Activity Promotes Hepatoma Cell Resistance to Proteasome Inhibitor Bortezomib
Source: Cancers (Basel). 2021 May 12;13(10):2341. doi: 10.3390/cancers13102341 (PMC8150958; doi:10.3390/cancers13102341)

**A**

**Proteasome Complex  
(GO:0000502)**

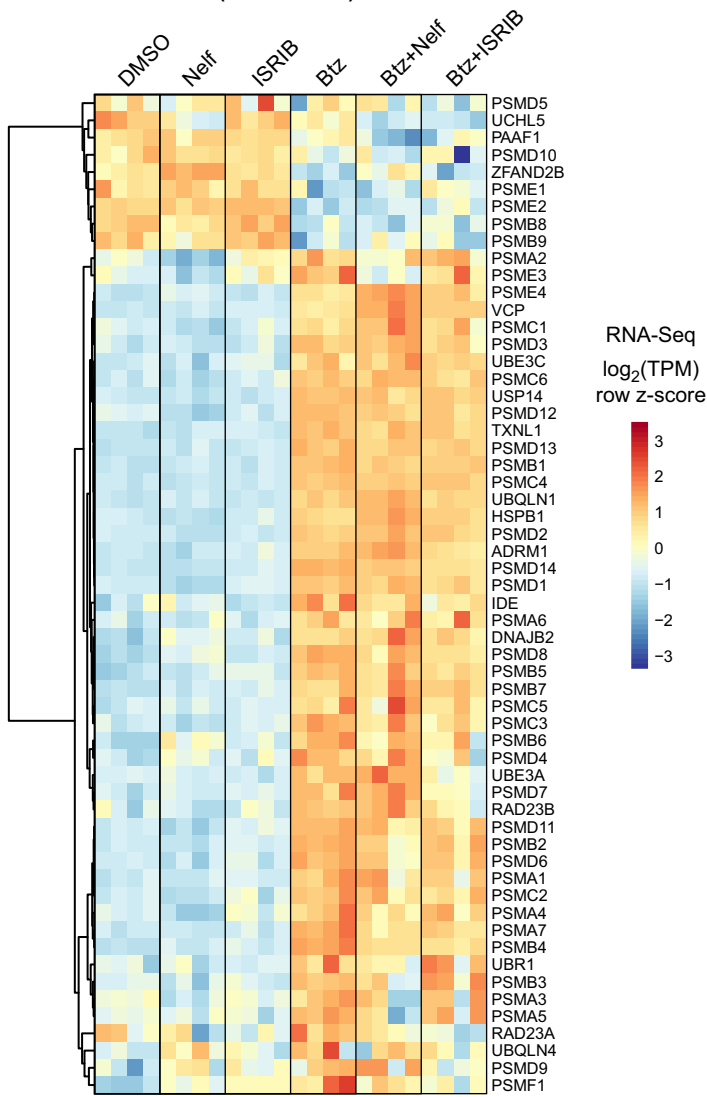**B**

**Gene set activity  
(Proteasome Complex GO:0000502)**

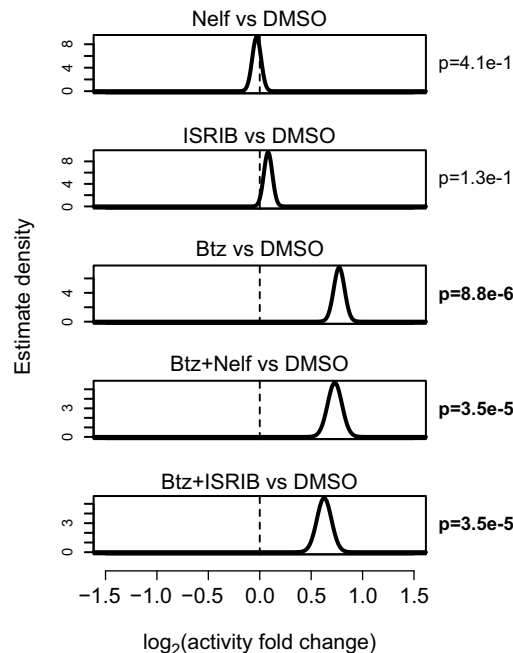

Supplement: Supplementary file 1 [file cancers-13-02341-s001.zip › Supplementary Figure S1.pdf]

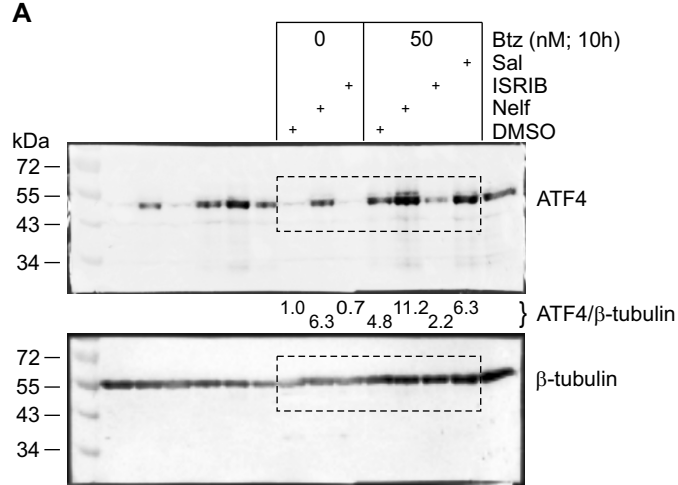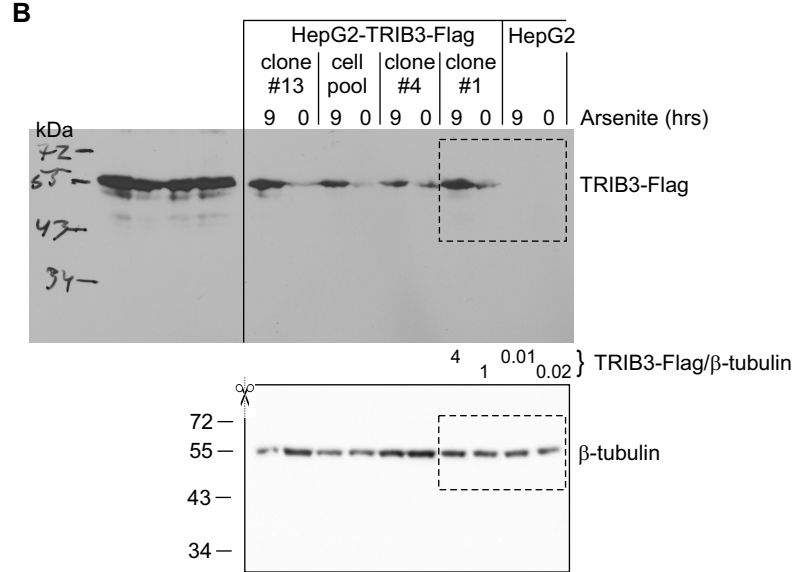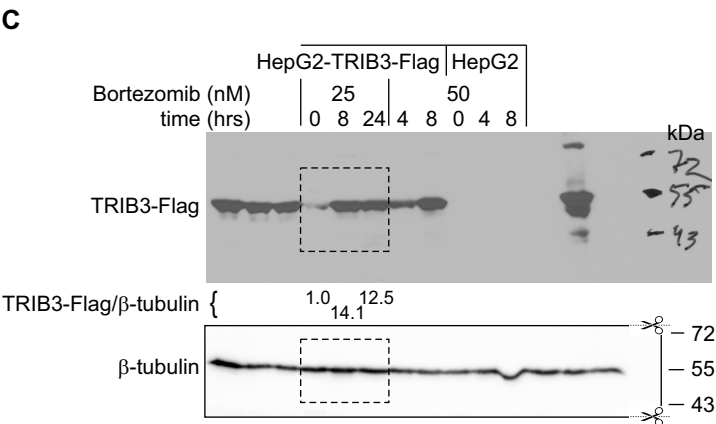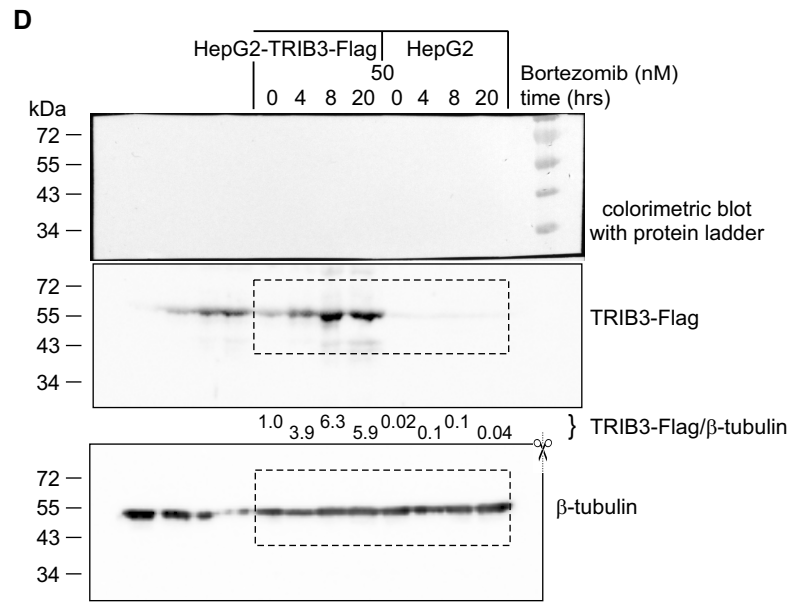

Supplement: Supplementary file 1 [file cancers-13-02341-s001.zip › Supplementary Figure S2.pdf]

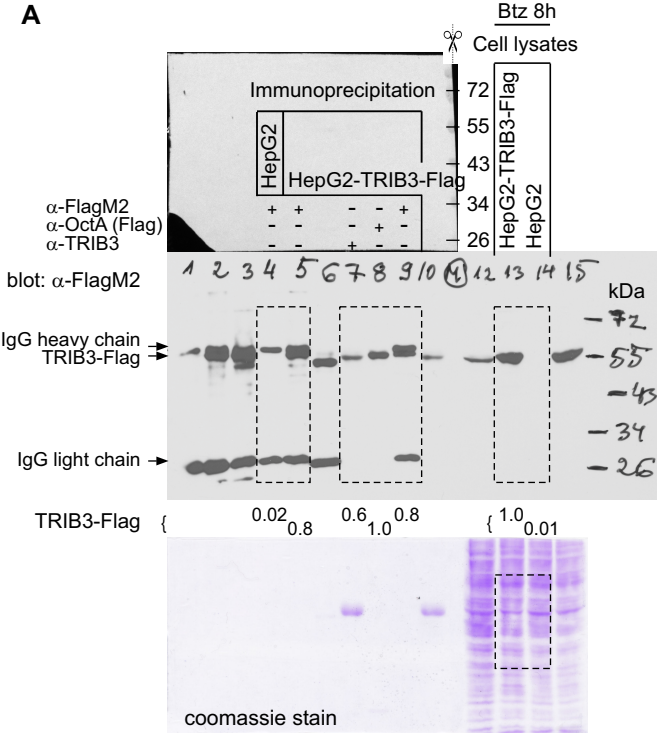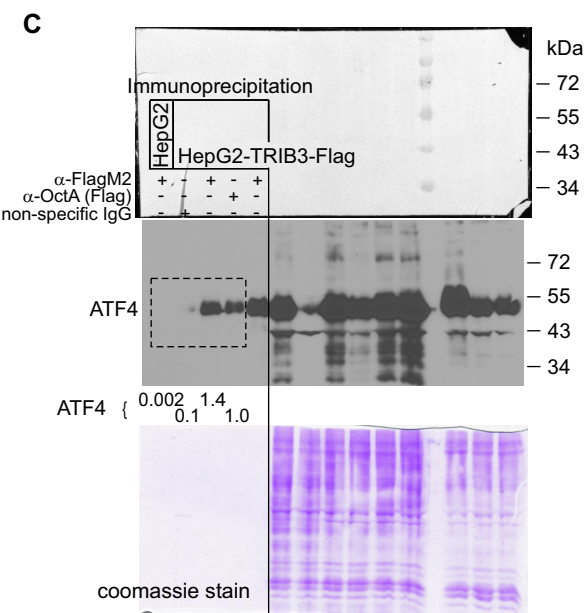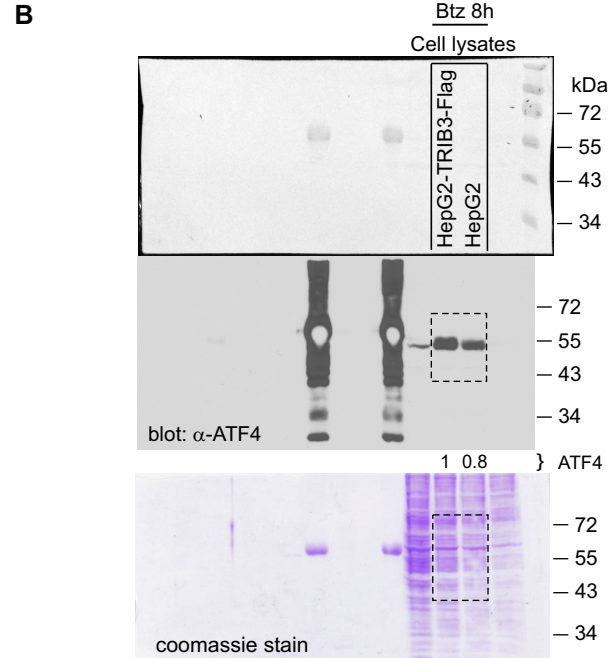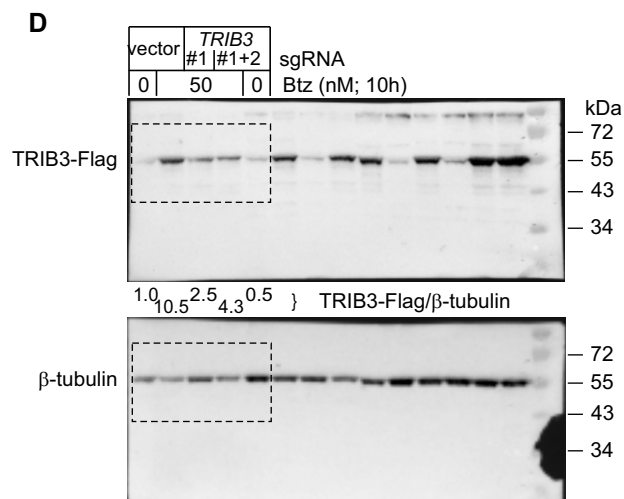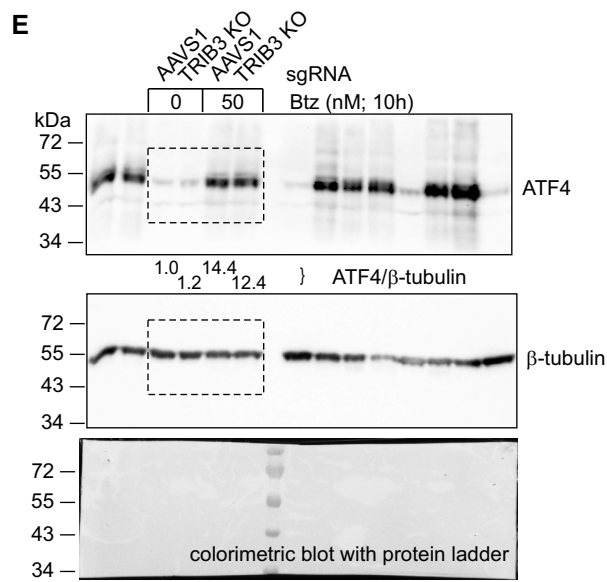

Supplement: Supplementary file 1 [file cancers-13-02341-s001.zip › Supplementary Figure S3.pdf]
